# Heat Shock Protein SSA1 Enriched in Hypoxic Secretome of *Candida albicans* Exerts an Immunomodulatory Effect via Regulating Macrophage Function

**DOI:** 10.3390/cells13020127

**Published:** 2024-01-10

**Authors:** Wei Teng, Phawinee Subsomwong, Kouji Narita, Akio Nakane, Krisana Asano

**Affiliations:** 1 Department of Microbiology and Immunology, Hirosaki University Graduate School of Medicine, Hirosaki 036-8562, Japan; h20gm132@hirosaki-u.ac.jp (W.T.); phawinee@hirosaki-u.ac.jp (P.S.); 2 Insititue for Animal Experimentation, Hirosaki University Graduate School of Medicine, Hirosaki 036-8562, Japan; nari@hirosaki-u.ac.jp; 3 Department of Biopolymer and Health Science, Hirosaki University Graduate School of Medicine, Hirosaki 036-8562, Japan; a27k03n0@hirosaki-u.ac.jp

**Keywords:** *Candida albicans*, hypoxia, secretome, heat shock protein SSA1, immunomodulation

## Abstract

*Candida albicans* is an opportunistic pathogenic yeast that can survive in both normoxic and hypoxic environments. The involvement of *C. albicans* secretome on host biological processes has been demonstrated. However, the immunoregulatory function of *C. albicans* secretome released under hypoxic condition remains unclear. This study demonstrated the differences in cytokine responses and protein profiles between secretomes prepared under normoxic and hypoxic conditions. Furthermore, the immunoregulatory effects of heat shock protein SSA1(Ssa1), a protein candidate enriched in the hypoxic secretome, were investigated. Stimulation of mouse bone marrow-derived macrophages (BMMs) with Ssa1 resulted in the significant production of interleukin (IL)-10, IL-6, and tumor necrosis factor (TNF)-α as well as the significant expression of M2b macrophage markers (CD86, CD274 and tumor necrosis factor superfamily member 14), suggesting that *C. albicans* Ssa1 may promote macrophage polarization towards an M2b-like phenotype. Proteomic analysis of Ssa1-treated BMMs also revealed that Ssa1 reduced inflammation-related factors (IL-18-binding protein, IL-1 receptor antagonist protein, OX-2 membrane glycoprotein and cis-aconitate decarboxylase) and enhanced the proteins involved in anti-inflammatory response (CMRF35-like molecule 3 and macrophage colony-stimulating factor 1 receptor). Based on these results, we investigated the effect of Ssa1 on *C. albicans* infection and showed that Ssa1 inhibited the uptake of *C. albicans* by BMMs. Taken together, our results suggest that *C. albicans* alters its secretome, particularly by promoting the release of Ssa1, to modulate host immune response and survive under hypoxic conditions.

## 1. Introduction

*Candida albicans* is a prevalent strain of yeast that can be detected in approximately 50% of the population. It is considered a normal constituent of the human microbiome, predominantly present in the oral, gastrointestinal, and vaginal regions in the human body [1]. However, it can lead to the development of illnesses, including superficial infections such as oral or vaginal candidiasis, as well as life-threatening systemic diseases. The development of infections usually occurs due to dysbiosis of normal microbiota, immune dysfunction, and damage of the mucosal barrier [2]. Life-threatening systemic infections of *C. albicans* cause a serious clinical problem, particularly among immunosuppressed individuals [1].

The interplay between *C. albicans* and host immune response is complex [3]. Macrophages play the important role of combating *C. albicans* infection [4]. They produce a large amount of tumor necrosis factors-alpha (TNF-α) and interleukin (IL)-6 in response to β-(1,3)-glucan, a major *C. albicans* cell wall component [5]. Although invading *C. albicans* can be eliminated rapidly by macrophages, *C. albicans* has evolved the mechanism to evade the host defense system [6]. Zheng et al. reported that *C. albicans* is able to induce the production of anti-inflammatory cytokines and block the conversion of macrophages from the M2 to the M1 phenotype [7]. It is known that classically activated M1 macrophages are critical for host defense against pathogens. They produce several proinflammatory cytokines, including TNF-α and IL-6 [8]. On the other hand, alternatively activated M2 macrophages produce anti-inflammatory cytokines such as IL-10 and are typically associated with tissue repair and remodeling [8]. Although the immunomodulatory effect of *C. albicans* via anti-inflammatory cytokines and M2 macrophages is suggested, the detailed mechanisms remain to be elucidated.

*C. albicans* secretes an array of proteins, including growth factors, extracellular matrix proteins, and enzymes, into secretome to facilitate interactions with host cells and tissues [9]. Previous studies have demonstrated that the proteins in the secretome of *C. albicans* have important effects on cellular behavior and exhibit many functions related to different biological processes [9,10,11,12,13,14]. For example, aspartic protease, a hydrolytic enzyme in *C. albicans* secretome facilitates invasion and nutrition intake of yeast cells and is also involved in host tissue degradation. We therefore hypothesize that some proteins in the *C. albicans* secretome may play an important role in regulating host immunity.

Human organs or tissues in a healthy state have a wide range of oxygen distribution, varying from less than 2% to a maximum of 14% [15,16]. The gastrointestinal system exhibits a physiologically hypoxic environment, from 2% to 8% oxygen, and less than 2% in the gut lumen [16,17]. Since *C. albicans* is a component of the resident microbiota in the human gastrointestinal tract [18], the ability of *C. albicans* to adapt to hypoxic environments is expected to be important for its survival and pathogenicity. However, there is limited understanding of the role of the secretome released from *C. albicans* under a hypoxic environment.

This study aims to elucidate the involvement of the *C. albicans* secretome released under hypoxic conditions on host immunomodulation. Immune responses to secretomes prepared under normoxic and hypoxic conditions were compared. One protein enriched in the hypoxic secretome that modulates the host inflammatory response was identified. Its functions contributing to cytokine production, macrophage polarization, and the pathogenicity of *C. albicans* were investigated.

## 2. Materials and Methods

### 2.1. C. albicans Strain and Cell Culture

*C. albicans* NBRC 1385 strain was cultured on yeast extract-peptone-dextrose (YPD; 1% yeast extract, 2% peptone, 2% dextrose) agar plate and incubated at 30 °C for 24 h before use. Mouse macrophage RAW 264.7 cells were cultured in Dulbecco’s Eagle’s minimum essential medium (Nissui Pharmaceutical Co., Ltd., Tokyo, Japan) supplemented with 10% fetal bovine serum (FBS; JRH Biosciences, Lenexa, KS, USA), 0.075% NaHCO_3_ (Wako Pure Chemical Industries, Osaka, Japan), 0.03% L-glutamine (Wako Pure Chemical Industries), and 1 × Antibiotic-Antimycotic (Gibco; ThermoFisher, Waltham, MA, USA) at 37 °C, 5% CO_2_.

### 2.2. Preparation of Normoxic Secretome (NS) and Hypoxic Secretome (HS) from C. albicans

*C. albicans* was inoculated at OD_600nm_ = 0.1 into YPD medium and cultivated at 30 °C. To prepare HS, yeast cells were grown under low oxygen conditions (filled medium in tightly closed 1000 mL-bottle with static condition) for 48 h, while cultivation under vigorous aeration (130 rpm agitation) for 16 h was used to prepare NS. It should be noted that the normoxic and hypoxic oxygen concentrations in uninoculated medium measured by a JPB-70A dissolved oxygen analyzer (Shen Zhen Yage Technology, Shenzhen, China) were 6.5 mg/L and 1.0 mg/L, respectively [19]. After removal of yeast cells by centrifugation and filtration through 0.45 μm filters, the secreted proteins in the supernatant were precipitated by ultracentrifugation at 99,800× *g*, 4 °C for 90 min, washed twice and dissolved in 1 × phosphate-buffered saline (PBS) buffer [19]. The proteins in secretome were filtrated using a 0.45 μm filter and kept at −80 °C until use. Protein concentration of the secretomes was evaluated using Bio-Rad Protein Assay Dye reagent (BIO-RAD, Hercules, CA, USA). To observe the protein pattern, 5 μg protein of each secretome was applied to 10% sodium dodecyl-sulfate polyacrylamide gel electrophoresis (SDS-PAGE) and stained using Silver Stain II Kit (Wako Pure Chemical Industries). Yeast cell lysates (10 μL each) prepared by disrupting yeast cells with 0.5 mm-diameter glass beads using a Micro Smash^TM^ MS-100R machine (Tomy Digital Biology Co., Ltd., Tokyo, Japan) were applied to 10% SDS-PAGE.

### 2.3. Mice and Ethical Statement

8–12 week old female BALB/c mice were purchased from CLEA Japan, Inc. (Tokyo, Japan) and housed under specific pathogen-free conditions at the Institute for Animal Experimentation, Hirosaki University Graduate School of Medicine with a temperature-controlled room (22 ± 2 °C) on a 12 h light-dark cycle. They were allowed ad libitum access to drinking water and basal diet (CE-2; CLEA Japan, Inc.). All animal experiments were conducted in accordance with the Animal Research Ethics Committee, Hirosaki University Graduate School of Medicine, and followed the Guidelines for Animal Experimentation, Hirosaki University (Permit number: AE01-2023-175). Mice were anesthetized using a mixture of 0.3 mg/kg medetomidine, 4 mg/kg midazolam, and 5 mg/kg butorphanol and sacrificed via cervical dislocation.

### 2.4. Isolation of Mouse Spleen Cells

Spleens were collected from BALB/c mice and squeezed in Roswell Park Memorial Institute 1640 medium (RPMI 1640; Nissui Pharmaceutical Co., Ltd.) supplemented with 10% FBS, 0.075% NaHCO_3_, 0.03% L-glutamine, and 1 × Penicillin-Streptomycin (Gibco). Spleen cells were filtered through stainless steel mesh (100 μm pore size) and collected by centrifugation at 500× *g*, 4 °C for 10 min. Erythrocytes were lysed by incubation with 0.85% NH_4_Cl (Wako Pure Chemical Industries) for 5 min. The spleen cells were then washed twice and resuspended in RPMI 1640 medium for further use.

### 2.5. Preparation of Mouse Bone Marrow-Derived Macrophages (BMMs)

Femurs and tibias were collected from BALB/c mice. After removal of the epiphyses, the bone marrow cells were flushed out with RPMI 1640 medium and filtered through a 100-μm stainless steel mesh. The cells were harvested by centrifugation at 200× *g*, 4 °C for 5 min, and resuspended in 0.85% NH_4_Cl. After incubation for 5 min, the cells were collected by centrifugation and washed twice with RPMI 1640 medium. The cells were then seeded into a 75 cm^2^ cell culture flask and cultured at 37 °C, 5% CO_2_ in RPMI 1640 medium supplemented with 10 ng/mL macrophage colony-stimulating factor (M-CSF; Wako Pure Chemical Industries). On Day 4, half volume of the culture supernatant was replaced with fresh RPMI 1640 medium containing 10 ng/mL M-CSF. On Day 8, the adherent cells were harvested and counted for further use.

### 2.6. Differential Proteomic Analysis of C. albicans NS and HS

Differential proteomic analysis between *C. albicans* NS and HS from three independent preparations was performed in triplicate by liquid chromatography-tandem mass spectrometry (LC-MS/MS) [19]. Briefly, proteins in the samples were precipitated by adding a fourfold volume of acetone and placing them at −20 °C for 2 h. The precipitated proteins were denatured with 50% trifluoroethanol and proteins were quantified by the BCA method. Twenty μg of proteins were used to alkylate cysteine residues by reduction and alkylation with iodoacetamide. Alkylated protein samples were fragmented into peptides by trypsin, and the peptides were desalted using MonoSpin C18 columns (GL Sciences Inc., Torrance, CA, USA). LC-MS/MS was performed using a nanoLC system (Eksigent 400, AB Sciex, Framingham, MA, USA) with a nano C18 reverse-phase capillary tip column (75 μm × 125 mm, 3 μm, Nikkyo Technos CO., Ltd., Tokyo, Japan) connected online to a mass spectrometer (TripleTOF 6600, AB Sciex). The mass spectrometer was operated in information-dependent acquisition (IDA) and data-independent acquisition (SWATH) mode for secretome proteome. Acquired spectra were searched against the UniProt reviewed database using the ProteinPilot 5.0.1 software (AB Sciex). The resulting group file was loaded into PeakView (v2.2.0, AB Sciex) as a library and peaks from SWATH runs were extracted with a false discovery rate (FDR) <1%. The SWATH files were then exported to the MarkerView software program (version 1.3.0.1; AB Sciex) and the peak areas of individual proteins were normalized to the sum of the peak areas of all detected proteins. The proteomic data of NS and HS are available using accession number PXD046763 and JPST002382 for ProteomeXchange and jPOST Repository, respectively. UniProt (https://www.uniprot.org/, accessed on 10 December 2021) and Candida Genome Database (http://www.candidagenome.org/, accessed on 10 December 2021) [20] were used for bioinformatic analysis. Volcano plot was constructed using Python 3.9. Gene Oncology (GO) and Kyoto Encyclopedia of Genes and Genomes (KEGG) pathways enrichment analysis was conducted to determine the functions of each protein using ClueGo add-in of Cytoscape 3.8.2.

### 2.7. Preparation of Recombinant Ssa1

The full length of *SSA1* gene was amplified by PCR using the genomic DNA of *C. albicans* NBRC 1385 as template. The sequences of forward and reverse primers containing NdeI and BamHI recognition site (underlined) are 5′-GCCATATGTCTAAAGCTGTTGGTATT-3′ and 5′-GCGGATCCTTAATCAACTTCTTCAACAGTT-3′, respectively. AmpliTaq Gold DNA polymerase (Applied Biosystems; ThermoFisher, Waltham, MA, USA) and the following thermal protocol were used to amplify *SSA1* gene: 95 °C for 10 min; 40 cycles of 95 °C for 1 min, 50 °C for 40 s, and 72 °C for 2 min; 72 °C for 10 min. The PCR product (1971 bp in size) was ligated into NdeI and BamHI-digested pET-15b vector (Novagen, Darmstadt, Germany) and transformed into *Escherichia coli* Rosetta (DE3) (Novagen). Protein expression was induced by 1 mM isopropyl-β-D-thiogalactopyranoside (Wako Pure Chemical Industries), and (His)6-Ssa1 fusion protein was purified using TALON^®^ Metal Affinity Resin (Takara, Tokyo, Japan), according to the manufacturer’s protocol. Lipopolysaccharide was removed from the purified protein using ProteoSpin Endotoxin Removal Mini Kit (Norgen Biotek Corp., Thorold, ON, Canada). The purified protein was confirmed by 8% SDS-PAGE. (His)6-tagged protein from the backbone pET-15b plasmid was purified by the similar method and used as control. The protein concentration was evaluated using Bio-Rad Protein Assay Dye reagent (BIO-RAD).

### 2.8. Cytokine Assay

RAW 264.7 cells, mouse spleen cells, and mouse BMMs were seeded in 24-well or 96-well culture plates and incubated with secretomes or recombinant Ssa1 for 24–48 h. The production of IL-10, IL-6 and TNF-α in the culture supernatant was quantified using commercial mouse enzyme-linked immunosorbent assay kits (Invitrogen; ThermoFisher, Carlsbad, CA, USA), according to the manufacturer’s protocols.

### 2.9. Effect of Ssa1 on Mouse Macrophages Viability

RAW 264.7 cells or BMMs (1 × 10^4^ cells/well) were seeded in a 96-well plate and incubated with or without recombinant Ssa1 (0.5 μg/well). After 24 h of incubation, the supernatant in each well was replaced with 100 μL fresh medium and 10 μL cell proliferation reagent (WST-1; Roche, Mannheim, Germany). After color development, the absorbance at 450 nm was measured using a microplate reader Multiskan Sky Microplate Spectrophotometer (Thermo Scientific™, Carlsbad, CA, USA). Cell-free medium with 10 μL cell proliferation reagent WST-1 was used as blank, and the absorbance at 600 nm was used as the reference wavelength.

### 2.10. Differential Proteomic Analysis of Ssa1-Treated and Untreated BMMs

Mouse BMMs (1 × 10^6^ cells/well) were seeded in a 24-well plate and incubated with or without recombinant Ssa1 (50 μg/well). At 24 h of incubation, the cells were washed with ice-cold 1 × PBS and lysed with lysis buffer (50 mM Tris pH 8.0, 150 mM NaCl, 1% NP40, 0.5% sodium deoxycholate). Differential proteomic analysis from three independent cell preparations was performed in triplicate by LC-MS/MS as above [19]. A DIA-NN software version 1.8.1 [21] was used to extract quantitative data for proteins from the SWATH runs with a library-free workflow. A predicted library from a UniProt mouse database was built in silico with the options ‘FASTA digest for library-free search/library generation’ and ‘Deep learning-based spectra, RTs and IMs prediction’. Quantitative data were output using RT-dependent cross-run normalization and filtering with a 1% FDR threshold. All other settings were left at default. Principal component analysis of the proteome data confirmed that there were no outlier proteomes in any of the samples. The proteomic data of Ssa1-treated and untreated BMMs are available using accession number PXD046766 and JPST002383 for ProteomeXchange and jPOST Repository, respectively. To determine the functions of proteins, GO enrichment analysis was conducted as described above.

### 2.11. Gene Expression Analysis of Ssa1-Treated BMMs

Mouse BMMs were incubated with or without recombinant Ssa1 as described above. After 24 h of incubation, total RNA was extracted using Trizol^®^ reagent (Life Technologies, Inc., Carlsbad, CA, USA) according to the manufacturer’s instructions. cDNA was synthesized using M-MLV Reverse Transcriptase (Invitrogen) and a random primer. The expression of macrophage-related genes was quantified using SYBR Green (BIO-RAD)-based detection with the CFX96™ Real-Time System (BIO-RAD). The primers for gene expression are listed in Table S1. The following thermal conditions were used: 95 °C for 5 min; 45 cycles of 95 °C for 10 s, 65 °C for 1 min. Gene expressions were normalized using the expression level of glyceraldehyde-3-phosphate dehydrogenase (GAPDH), and the results were expressed as relative values.

### 2.12. Construction of Ssa1-Deficient Mutant of C. albicans (Ssa1^−^)

Ssa1^−^ was constructed by the CRISPR/Cas9 method [22] with some modifications. Briefly, guide RNA was designed from protospacer adjacent motif sequence in *SSA1* gene [23]. DNA oligomers for guide RNA were annealed, ligated into Esp3I-digested pV1524 plasmid (Gerald Fink, Cambridge, MA, USA), and transformed into *E. coli* DH5α. Repair template containing stop codon and EcoRI recognition site was designed. PCR fragments of repair template were prepared, combined, and cloned into pCR2.1 plasmid. Correct sequences for guide RNA and repair template were confirmed by DNA sequencing. Linearized guide RNA (KpnI/SacI) and repair template (HindIII/XhoI) were transformed into *C. albicans*, which were prepared as competent cells by polyethylene glycol 3350/LiAc method. From the nourseothricin resistant colonies, PCR was performed to screen for the *SSA1* gene mutation, whose PCR product could be cleaved by EcoRI. The colonies with *SSA1* gene mutation were repeatedly sub-cultured in Yeast Peptone Maltose medium. *SSA1* gene mutation from nourseothricin-susceptible colonies was confirmed by sequencing. The related oligomers for Ssa1^−^ construction are listed in Table S2. Lack of Ssa1 protein in the Ssa1^−^ was confirmed by Western blot using anti-Ssa1 antibodies in mouse serum produced in our laboratory.

### 2.13. Infection of Ssa1-Preteated BMMs with C. albicans

Mouse BMMs (1 × 10^4^ cells/well) were incubated with or without recombinant Ssa1 (0.5 μg/well) for 24 h and infected with *C. albicans* wild-type or Ssa1^−^ mutant at 1:1 multiplicity of infection. After 3 h of infection, extracellular yeast cells were collected and enumerated by plate count method.

### 2.14. Statistical Analysis

Data management and statistical analysis were performed using Prism9 software (Version 9.5.0). The method for statistical analysis was mentioned in each figure legend. A *p*-value < 0.05 was considered to be statistically significant.

## 3. Results

### 3.1. Cytokine Production in Mouse Macrophages and Spleen Cells Induced by C. albicans NS and HS

**Figure 1:**
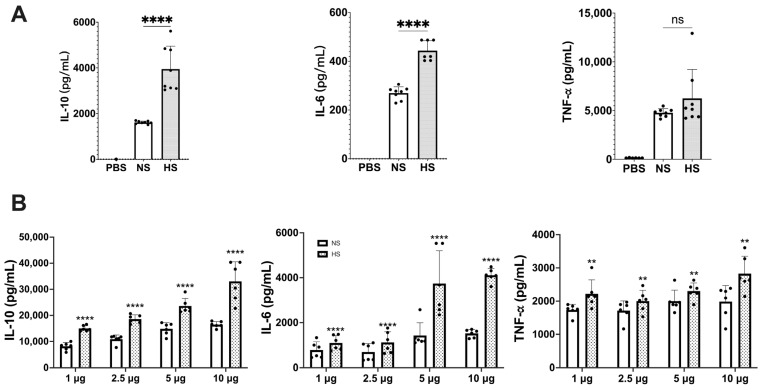
Cytokine responses in (**A**) mouse macrophages and (**B**) mouse spleen cells stimulated by NS and HS. (**A**) RAW 264.7 cells (1 × 10^6^ cells/well) were incubated with 5 μg protein of NS or HS. After 48 h of stimulation, IL-10, IL-6 and TNF-α production in culture supernatant was determined by ELISAs (ns: not significant, ****: *p* < 0.0001, statistical analysis by unpaired Student’s *t*-test, *n* = 8 from 2 independent experiments). (**B**) Mouse splenocytes (5 × 10^6^ cells/well) were incubated with 1 μg, 2.5 μg, 5 μg, and 10 μg of NS or HS. After 48 h of stimulation, IL-10, IL-6, TNF-α production in culture supernatant was determined by ELISAs. The IL-10, IL-6 and TNF-α levels between NS and HS have significant differences (*p* < 0.0001, statistical analysis by two-way ANOVA, *n* = 6 from two independent experiments). Each dose between NS and HS in IL-10, IL-6, and TNF-α has significant differences (**: *p* < 0.0021, ****: *p* < 0.0001, statistical analysis by Tukey’s test).

From 4 L of *C. albicans* NBRC 1385 culture medium, the total number of yeast cells under normoxic and hypoxic conditions was 4.3 × 10^10^ CFU and 1.58 × 10^10^ CFU, yielding 1.25 and 0.6 mg of NS and HS protein, respectively. Differences between NS and HS protein patterns were also observed (Figure S1). To investigate potential differences in immunoregulatory functions between NS and HS, the levels of IL-10, IL-6, and TNF-α production in RAW 264.7 and mouse spleen cells were assessed. As shown in Figure 1A, HS significantly stimulated the production of IL-10 and IL-6 compared to NS, whereas the TNF-α production between the two groups was comparable. By using mouse spleen cells and various doses of secretome protein, HS significantly stimulated the production of IL-10, IL-6, and TNF-α compared to NS (Figure 1B). The differences in TNF-α production between RAW 264.7 and spleen cells might be due to the fact that the spleen cells are associated with many types of immune cells those influence TNF-α production.

### 3.2. Differential Proteomic Analysis between NS and HS of C. albicans

**Figure 2:**
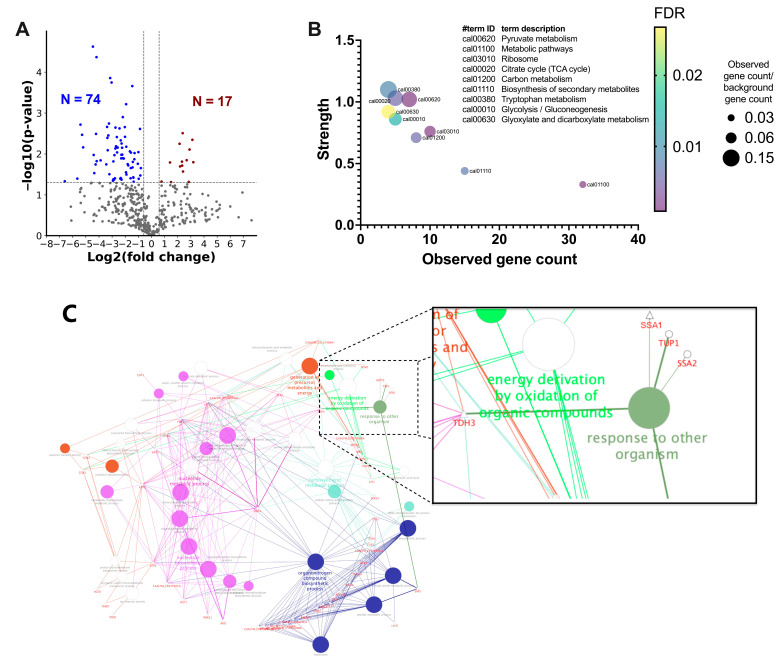
Differential proteomic analysis between NS and HS. (**A**) Volcano plot for comparing the differences in NS and HS proteins. The *x*-axis represents the log2 of fold-change value (HS/NS), whereas *y*-axis shows the −log10 of *p*-value. The cutoff criteria, represented by the dash lines were |log2FC| ≥ 0.585 and −log10(*p*-value) > 1.3 (*p* < 0.05, Student’s *t*-test). Blue dots: proteins abundant in NS (N = 74); red dots: proteins abundant in HS (N = 17). (**B**) KEGG enrichment analysis for proteins abundant in NS. The results revealed that most of these proteins were involved in metabolic pathways. (**C**) GO term and KEGG pathway enrichment analysis of the significantly differential proteins revealed that the function of Ssa1, Tup1, and Ssa2 is associated with response to other organisms (GO:0051707), including the terms of defense response to other organism (GO:0098542), detection of other organisms (GO:0098543), response to host (GO:0075136), and response to defenses of other organisms (GO:0052173). ○: proteins abundant in NS; △: proteins abundant in HS. A higher resolution of Figure 2B,C is provided in Figures S4 and S5, respectively.

To compare the difference in protein compositions between NS and HS, a quantitative proteomic analysis was performed. A total of 490 proteins were quantified (Table S3). With a threshold of |log2FC| ≥ 0.585 and −log10(*p*-value) > 1.3, 74 proteins in HS were less abundant than those in NS (Figure 2A; Table S3). According to the KEGG database, most of these proteins are involved in metabolic pathways (Figure 2B). In addition, 17 proteins were enriched in HS (Figure 2A; Table 1). GO and KEGG pathway enrichment analysis of the proteins with significant differences revealed that heat shock protein SSA1 (Ssa1), heat shock protein SSA2 and transcriptional repressor TUP1 are involved in the process of responding to other organisms (Figure 2C). Of these, Ssa1, a chaperonin in the Hsp70 family, is the only protein that was upregulated in HS. Based on the immunoregulatory properties of Hsp70 [24], we hypothesized that Ssa1 might be a protein that modulates host immune responses, especially when *C. albicans* is in a hypoxic environment.

### 3.3. Effect of Ssa1 on Cytokine Response and Viability of RAW 264.7 Cells

To elucidate the role of *C. albicans* Ssa1 on the host immune response, recombinant Ssa1 was prepared by a (His)6-tagged fusion system, and lipopolysaccharide (LPS) was removed (Figure S2A). Unfortunately, the His-tag could not be removed from the recombinant protein due to the presence of an unexpected thrombin-cleavage site in Ssa1. Therefore, to confirm that neither the His-tag nor residual LPS affect the immune response, (His)6-tagged protein from the pET-15b backbone was prepared by the same method and used as a control. As shown in Figure S2B, IL-10 and IL-6 production was observed only in (His)6-tagged Ssa1 but not the (His)6-tagged control protein. These results indicate that Ssa1 induces cytokine production, and that neither the His-tag nor LPS interfered with these cytokine responses.

The effect of Ssa1 on RAW 264.7 cell viability was also examined. As shown in Figure S2C, there was no statistically significant difference in cell viability between Ssa1-treated and untreated groups, suggesting that *C. albicans* Ssa1 is not toxic to RAW 264.7 cells.

### 3.4. Cytokine Production and Protein Expression in Ssa1-Treated BMMs

**Figure 3:**
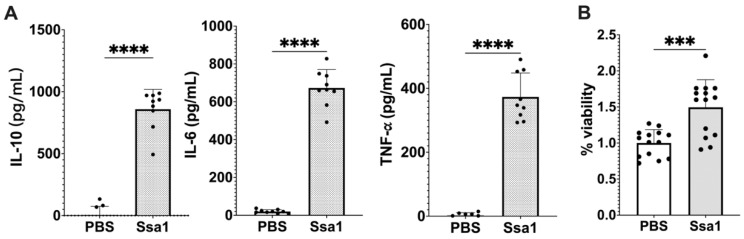
Effect of *C. albicans* Ssa1 on (**A**) cytokine production and (**B**) viability of mouse BMMs. (**A**) BMMs (1 × 10^5^ cells) were seeded in 24-well plates and treated with or without 5 μg Ssa1. After 48 h of stimulation, the production of IL-10, IL-6, and TNF-α in the culture supernatant was determined by ELISAs. The production of IL-10, IL-6, and TNF-α in Ssa1-treated macrophages was significantly higher than that in the untreated control group. (****: *p* < 0.0001, statistical analysis by unpaired Student’s *t*-test, *n* = 9 from three independent experiments) (**B**) BMMs (1 × 10^4^ cells/well) were seeded in a 96-well plate and treated with or without 0.5 μg Ssa1. After 24 h of incubation, the culture supernatant in each well was replaced with 100 μL of fresh medium containing 10 μL of cell proliferation reagent (WST-1; Roche). After color development, the absorbance at 420 nm was measured using the absorbance at 600 nm as a reference wavelength. Cell-free medium was used as a blank, and the absorbance of BMMs without Ssa1 was calculated as 100% viability (***: *p* < 0.0002, statistical analysis by unpaired Student’s *t*-test, *n* = 14 from three independent experiments).

For the next experiments, BMMs were used because of the limitation that RAW 264.7 cells are not functional for certain signaling pathways [25]. The results showed that Ssa1 significantly stimulated the production of IL-10, IL-6, and TNF-α from BMMs (Figure 3A). Moreover, the effect of *C. albicans* Ssa1 on the cell viability of BMMs was conducted. As shown in Figure 3B, Ssa1 not only showed no cytotoxicity but also promoted BMM proliferation, indicating that the effect of *C. albicans* Ssa1 on cell viability differs between BMMs and RAW 264.7 cells.

**Figure 4:**
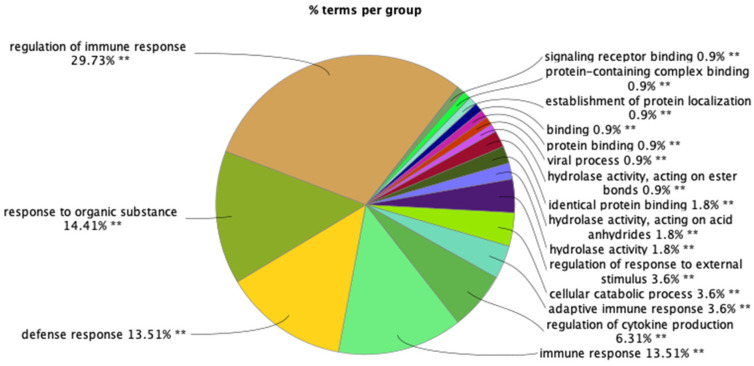
GO enrichment analysis of the significantly upregulated and downregulated proteins in Ssa1-treated BMMs. Main functions of the proteins are related to immune system processes (**: *p* < 0.001).

To further investigate the impact of *C. albicans* Ssa1 on BMM functions, the differential protein production between Ssa1-treated and untreated BMMs was analyzed. A total of 3704 proteins were relatively quantified (Table S4). Among these, 80 proteins showed significant differences between two groups (*q*-value less than 0.05) (Table 2). Seven proteins including interleukin-18-binding protein (IL18BP) and T-lymphocyte activation antigen CD86 were detected only in Ssa1-treated group. Sixty proteins significantly increased (1.113- to 6.697-fold change) and 13 proteins significantly decreased (0.398- to 0.844-fold change) after Ssa1 treatment. GO enrichment analysis revealed that main functions of these 80 proteins are related to immune system processes (Figure 4; Table S5).

### 3.5. Macrophage Polarization in Ssa1-Treated BMMs

**Figure 5:**
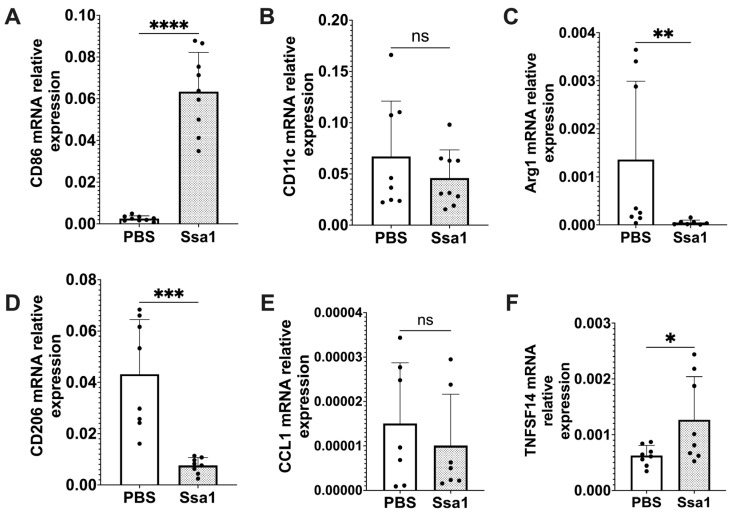
Relative mRNA expression of the markers associated with macrophage polarization in Ssa1-treated BMMs. BMMs (1 × 10^5^ cells) were seeded in 24-well plates and treated with or without 5 μg Ssa1. After 24 h of treatment, total RNA from the cells was extracted, and cDNA was synthesized and used as a template for RT-qPCR. mRNA levels were normalized according to the expression level of GAPDH. Results were expressed using the ΔΔCt method for quantitation. (**A**) CD86, (**B**) CD11c, (**C**) Arg1, (**D**) CD206, (**E**) CCL1 and (**F**) TNFSF14 (ns: not significant, *: *p* < 0.0332, **: *p* < 0.0021, ***: *p* < 0.0002, ****: *p* < 0.0001, statistical analysis by unpaired Student’s *t*-test, *n* = 8 from 3 independent experiments).

From the differential proteomic analysis results, CD86 was observed only in the Ssa1-treated BMMs with mean expression value of 6.15 (Table 2). Because CD86 is an important marker for M1 macrophages [26], we further investigated the expression of other macrophage markers to assess the impact of *C. albicans* Ssa1 on M1 and M2 polarization. As shown in Figure 5A, the mRNA expression of CD86 was significantly up-regulated in Ssa1-treated BMMs. However, the mRNA expression of CD11c, which is another M1 macrophage marker [8], was not altered (Figure 5B). In addition, significant decreases in the mRNA expression of Arginase-1 (Arg1) and CD206, which are commonly used as M2 macrophage markers [8], were observed in the Ssa1-treated BMMs (Figure 5C,D). From these results, it is unclear whether Ssa1 contributes to M1 or M2 macrophage polarization.

In addition to CD86, the proteomic analysis results in Table 2 showed that CD274 was also significantly up-regulated in the Ssa1-treated BMMs with fold change of 2.883 (Table 2). Regarding both CD86 and CD274 as important markers for M2b macrophages [26], we hypothesized that Ssa1 may be related to M2b macrophage polarization. Recently, an increasing number of studies have suggested that M2 macrophages are not a homogeneous population, but rather exhibit functional variety [26]. M2 macrophages have been believed to possess an anti-inflammatory role. However, M2b macrophages, classified as an M2 subtype, exhibit notable production of the anti-inflammatory cytokine, IL-10, as well as IL-6 and TNF-α simultaneously. Furthermore, M2b macrophages are more likely to express CD86 and CD274 [26], in contrast to Arg1 and CD206 which are commonly used as M2 macrophage markers [8]. To confirm our hypothesis regarding the effect of Ssa1 on M2b macrophage polarization, we evaluated the mRNA expression of C-C Motif Chemokine Ligand 1 (CCL1) and tumor necrosis factor superfamily member 14 (TNFSF14), which are secreted products of M2b macrophages. Although the expression of CCL1 was not promoted by Ssa1 (Figure 5E), the expression of TNFSF14 increased significantly in the Ssa1-treated BMMs (Figure 5F). Based on the up-regulation of CD86, CD274 and TNFSF14 (Table 2, Figure 5A,F), we therefore assumed that *C. albicans* Ssa1 promotes macrophage polarization into the M2b-like phenotype.

### 3.6. Ssa1 Inhibits the Uptake of C. albicans by BMMs

**Figure 6:**
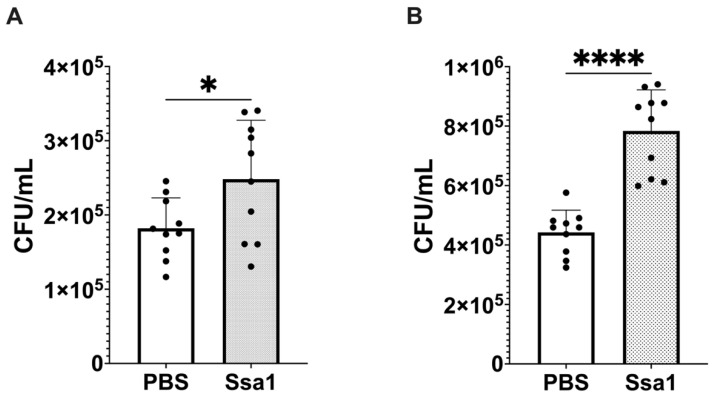
Ssa1 inhibits the uptake of *C. albicans* by BMMs. BMMs were pretreated with Ssa1 for 24 h and infected with (**A**) the wild-type strain of *C. albicans* NBRC 1385 and (**B**) Ssa1-deficient mutant of *C. albicans* NBRC 1385 (Ssa1^−^) at MOI = 1:1. At 3 h of infection, extracellular yeast cells were collected and enumerated by the plate count method. The data are expressed as colony-forming units (CFU)/mL. The statistical significance of the differences in CFU numbers was evaluated by using unpaired Student’s *t*-test (*: *p* < 0.0332; ****: *p* < 0.0001, *n* = 10 from 2 independent experiments).

As M2b macrophages release anti-inflammatory cytokines and play a role in promoting fungal infection, the involvement of Ssa1 in M2b-like macrophage polarization suggests that Ssa1 may interfere with the macrophage capability to eradicate fungal cells. Thus, infection experiments were conducted. BMMs were pretreated with Ssa1 24 h prior to *C. albicans* infection, and the number of *C. albicans* remaining in the supernatant was assessed at 3 h after infection. As shown in Figure 6A, the number of extracellular *C. albicans* in Ssa1-pretreated macrophages was higher than that in untreated control, suggesting that Ssa1 reduces the ability of BMMs to uptake *C. albicans*.

It has been known that Ssa1 is released into the secretome and also localizes in the cytoplasm and cell wall surface of yeast cells [1,27]. To circumvent the effects of cytoplasmic and cell wall Ssa1 on *C. albicans* infection, an Ssa1-deficient mutant (*C. albicans* Ssa1^−^) was constructed (Figure S3). Infection experiments with this mutant were also carried out in the same manner as the wild type. The results in Figure 6B also showed that Ssa1-pretreatment significantly reduced the ability of BMMs to uptake *C. albicans*.

## 4. Discussion

*C. albicans* is an aerophilic microorganism preferring oxygen for metabolism and growth. However, it can also adapt to survive under a hypoxic environment and exists in the human gastrointestinal tract [1]. Since the microorganisms are in constant existence with other species in their environment, the species with more efficient stress tolerance have a survival advantage. Guo and Gross reported that stress induces the remodeling of the proteomic profile in microorganisms [28]. Therefore, under hypoxic environment, *C. albicans* would be expected to secrete molecules that are important for survival, especially for modulating host immune responses.

As expected, HS remarkably stimulated anti-inflammatory rather than pro-inflammatory cytokines (Figure 1). These results prompt us to further compare the differences in the protein profile between NS and HS with a particular focus on the proteins abundant in HS (Table 1). Of these, endo-1,3-glucanase (Eng1), yeast-form wall protein 1 (Ywp1), glyceraldehyde-3-phosphate dehydrogenase (Tdh3), and heat shock protein SSA1 (Ssa1) have been reported to contribute to the immunoregulation and pathogenicity of *C. albicans* [9,29,30,31,32]. Eng1 belongs to the glycosyl hydrolases family and has 1,3-β-glucanase activity [33]. *Histoplasma* yeast lacking the *ENG1* gene increases cell wall β-glucan, improves binding to β-glucan receptor (Dectin-1), and enhances the production of cytokine such as IL-6 and TNF-α by macrophages and dendritic cells [31]. The level of β-glucan in *C. albicans* cell wall can be regulated by Eng1 and correlates with the colonization ability of *C. albicans* in the gastrointestinal tract [34,35]. Ywp1 is a yeast-specific wall protein that is not expressed on the hyphal surface [36] and insists on adhesion to facilitate the dispersal of yeast to colonize new sites [32]. Together with Eng1, Ywp1 regulates cell wall β-glucan levels [32] to prevent Dectin-1 recognition [31]. The function of intracellular Tdh3 and Ssa1 is related to the glycolysis pathway and chaperoning, respectively. However, Tdh3 and Ssa1 have been identified as cell wall-associated moonlighting proteins of *C. albicans* that are immunoreactive during invasive infection in humans [30]. In *C. albicans*, the cell wall-associated form of Tdh3 has a role in promoting fungal cell adhesion to fibronectin and laminin, which aids in *Candida* attachment to host tissues and infection propagation [37]. On the other hand, the cell wall-associated form of Ssa1 in *C. albicans* is involved in endocytic induction and acts as a receptor for the salivary histidine-rich basic antifungal peptide, histatin 5 [30,38]. Although these four proteins are candidates for further studies on immunomodulation and pathogenicity, GO and KEGG pathway enrichment analysis revealed that Ssa1 is the only protein enriched in HS that is involved in responding to the other organisms.

Ssa1 is a protein of the Hsp70 family. Hsp70 is an all-encompassing molecular chaperone that participates in numerous cellular stress responses. By attaching to exposed hydrophobic areas, Hsp70’s principal function is to impede the aggregation of denatured proteins and prevent the formation of amorphous aggregates [27]. Heat shock proteins including Ssa1 were first believed to localize intracellularly. However, they can also be released extracellularly and activate the immune system in response to current harmful cellular circumstances. Different Hsp species have different impacts on immunological cells [39,40]. In experimental disease models, the administration of Hsp has demonstrated the ability to hinder the occurrence of inflammatory damage. Furthermore, preliminary clinical trials in patients with chronic inflammatory diseases revealed that Hsp peptides can stimulate the production of anti-inflammatory cytokines. These findings suggest that Hsp possesses immunoregulatory capabilities [24]. Our results showed that *C. albicans* secretes a high amount of Ssa1 under hypoxia. Therefore, Ssa1 is expected to be an important extracellular factor that *C. albicans* releases to facilitate its evasion of the immune system, in particular, in hypoxic environments.

By using mouse BMMs, recombinant Ssa1 significantly enhanced the expression of macrophage markers CD86 and CD274 and stimulated IL-10, IL-6, and TNF-α production. IL-6 has long been widely considered a pro-inflammatory cytokine. However, it should be noted that it also possesses anti-inflammatory properties [41,42,43]. As shown in a previous study, IL-6 has the ability to reduce the intensity of acute inflammatory reactions [44] since it induces the production of TNF antagonists and IL-1RA by macrophages [45]. It has been observed that IL-6 has a direct effect on the polarization of macrophages towards the M2 phenotype [46]. In addition to CD86 and CD274, which are M2b macrophage markers, CCL1 and TNFSF14 are reliable indicators for identifying M2b macrophages [26]. Although our results showed that Ssa1 did not promote the expression of CCL1, it significantly enhanced the expression of TNFSF14. Taken together, the enhancement of IL-10, IL-6, TNF-α, CD86, CD274 and TNFSF14 in Ssa1-treated BMMs suggests that Ssa1 promotes macrophage polarization toward M2b-like phenotype (Figure 3A and Figure 5). It is important to note that the M2b phenotype is typically generated by exposure to immune complexes or Toll-like receptor (TLR) ligands. In our experiment, the BMMs were not exposed to these ligands. This may affect the expression of CCL1 [47]. From the results in Figure S3C and Figure 3B, Ssa1 was non-toxic to both RAW 264.7 cells and BMMs, and Ssa1 appeared to promote BMM proliferation compared to the untreated control (Figure S3C and Figure 3B). As our study used the succinate-tetrazolium reductase system to detect cell viability, these results may reflect the difference in metabolic activity between M1 and M2 macrophages [48].

In addition to the role of Ssa1 on M2b-like macrophage polarization, several proteins in BMMs that were significantly altered after Ssa1 treatment also imply the immunomodulatory effect of Ssa1. IL-18BP, cis-aconitate decarboxylase (ACOD1), OX-2 membrane glycoprotein (CD200), and IL-1 receptor antagonist protein (IL-1RA) were upregulated in Ssa1-treated BMMs (Table 2). IL-18BP has a notable affinity for binding IL-18 and thus modulates the functional activity of this cytokine [49,50,51]. The presence of IL-18BP downregulates T-helper 1 responses and thus reduces the induction of interferon-γ [52]. Joseph T et al. demonstrated that the inhibition of IL-18BP leads to an increased resistance to infections [53]. ACOD1, also known as IRG, is a mitochondrial enzyme capable of producing itaconate, which plays a regulatory role in inflammation by inhibiting the synthesis of cytokines and reactive oxygen species [54]. CD200 has the ability to reduce the production of TNF-α by macrophages [55,56]. The reduction in TNF-α production can lead to a decrease in the activity of the NF-κB transcription factor, ultimately resulting in the negative regulation of the macrophage lineage [57]. IL-1RA has been reported to interact with the IL-1 receptor. This interaction inhibits the binding of IL-1 to receptor, effectively halts the pro-inflammatory signaling, and attenuates the inflammatory effects of IL-1 [58,59,60,61]. In addition to these upregulated proteins, CMRF35-like molecule 3 (CD300LD3) and macrophage colony-stimulating factor 1 receptor (CSF1R) were downregulated in Ssa1-treated BMMs (Table 2). CD300LC3 can enhance the ubiquitination of TNF receptor-associated factor 6 and promote the release of TLR9-triggered cytokines [62]. The attenuation of TLR9 signaling in macrophages by a reduction of CD300LC3 enrichment has been shown to result in a decrease in the production of pro-inflammatory cytokines [62]. It has been observed that a reduction in CSF1R expression might lead to an enhanced signaling of IL-4 in macrophages in vivo and in vitro. This, in turn, contributes to the stimulation of M2-like macrophages with tissue repair phenotypes [63].

In addition to the proteins mentioned above, the proteomic analysis of Ssa1-treated BMMs showed that radical S-adenosyl methionine domain-containing protein 2 (RSAD2) was significantly up-regulated, and collectin-12 (COLEC12) was significantly down-regulated. RSAD2 can facilitate T cell receptor-mediated GATA-3 activation and optimize Th2 cytokine production by regulating NF-κB/p50 [64]. A number of observations suggests that NF-κB/p50 may be a key modulator for M2b macrophage polarization [65,66,67,68]. It is widely believed that M2b polarization is associated with accelerated infection. A previous study showed that M2b macrophages inhibit the differentiation of macrophages from M0 to M1, thereby reducing anti-pathogen activity [26]. The presence of M2b macrophages at the site of infection is associated with increased susceptibility to gastrointestinal candidiasis [69]. Kobayashi et al., showed that M2b macrophages play an important role in reducing infection resistance in mice, leading to bacterial translocation and eventual sepsis [70]. On the other hand, COLEC12 has been characterized as a scavenger receptor C-type lectin, a pattern recognition protein belonging to the innate immune system [71], and the main function of COLEC12 is to mediate the phagocytosis of fungi [72,73]. Based on these studies, the effect of Ssa1 on *C. albicans* uptake by BMMs was examined, and the results showed that Ssa1 pre-treatment reduced the uptake capability of BMMs towards *C. albicans*. A limitation of our experiments is that we assessed extracellular *C. albicans* rather than the intracellular population. In fact, assessing intracellular *C. albicans* in the macrophages is a more accurate method for this experiment. Unfortunately, we were unable to attain the antifungal agents suitable for eliminating only extracellular *C. albicans* and that adhered to the macrophage surface. Taken together, our results clearly demonstrated that *C. albicans* under hypoxic stress significantly releases extracellular Ssa1 to promote M2b-like macrophage polarization, modulate inflammation and immune responses, and facilitate the survival of extracellular *C. albicans* by attenuating uptake by macrophages. These findings provide a better understanding of *C. albicans* adaptation and its pathogenicity that may lead to new strategies for prevention of *C. albicans* infection, in particularly under hypoxic environment.

## 5. Conclusions

In this study, we demonstrated the differences in the protein composition of *C. albicans* secretomes released under normoxic and hypoxic environments. In particular, Ssa1, a protein abundant in the hypoxic secretome, was shown to promote M2b-like macrophage polarization, modulate inflammatory responses and reduce the ability mouse BMMs to phagocytose *C. albicans*. These findings suggest that *C. albicans* modifies its secretome in order to enhance its survival under hypoxic environments.

## Figures and Tables

**Table 1:** List of proteins that are enriched in HS with significant differences.

Gene ID	Protein Name	*p*-Value	Fold Change (Hypoxic vs. Normoxic)	Mass (Da)
*orf19.1672*	Coatomer subunit alpha	0.01127	Infinity	104,312
*MLC1*	Mlc1p	0.03923	Infinity	16,444
*RPL18*	Ribosomal 60S subunit protein L18A	0.04962	Infinity	20,770
*MUQ1*	CTP:phosphoethanolamine cytidylyltransferase	0.01592	9.134821103	42,013
*ACT1*	Actin	0.00451	8.696141484	41,690
*ENG1*	Endo-1,3(4)-beta-glucanase 1	0.00781	7.471613819	124,051
*SER33*	Phosphoglycerate dehydrogenase	0.04826	7.18653572	50,359
*YWP1*	Yeast-form wall Protein 1	0.01423	6.437111487	54,278
*RNR21*	Ribonucleotide-diphosphate reductase subunit	0.02691	5.305513281	47,437
*HSP104*	Chaperone ATPase	0.00311	5.208629718	99,919
*RPL8B*	60S ribosomal protein L8	0.01923	5.078615072	28,498
*GCA2*	Gca2p	0.01544	5.017964135	105,678
*TDH3*	Glyceraldehyde-3-phosphate dehydrogenase	0.02019	4.544732628	35,833
*EVP1*	Uncharacterized protein	0.00565	4.418480132	44,929
*orf19.1626*	Deoxyhypusine synthase	0.0487	2.815387167	41,634
*SSA1*	Heat shock protein SSA1	0.01616	2.66623664	70,324
*YPT1*	Rab family GTPase	0.04738	1.705239723	23,019

**Table 2:** List of proteins differentially expressed in Ssa1-treated and untreated bone marrow-derived macrophages with *q*-value less than 0.05.

Gene	Protein	Mean (Control)	Mean (Ssa1)	*q*-Value *	Fold Change
*Rsad2*	Radical S-adenosyl methionine domain-containing protein 2	0.000	47.557	0.001	
*Nmes1*	Normal mucosa of esophagus-specific gene 1 protein	0.000	13.452	0.027	
*Mov10*	Putative helicase MOV-10	0.000	9.290	0.027	
*Herc6*	E3 ISG15--protein ligase Herc6	0.000	9.179	0.002	
*Ifi205a*	Interferon-activable protein 205-A	0.000	7.494	0.014	
*Il18bp*	Interleukin-18-binding protein	0.000	6.862	0.005	
*Cd86*	T-lymphocyte activation antigen CD86	0.000	6.152	0.018	
*Cd40*	Tumor necrosis factor receptor superfamily member 5	3.189	42.712	0.000	6.697
*Cmpk2*	UMP-CMP kinase 2, mitochondrial	87.543	489.552	0.001	5.592
*Acod1*	Cis-aconitate decarboxylase	14.565	91.405	0.027	5.230
*Oasl1*	2′-5′-oligoadenylate synthase-like protein 1	13.636	65.679	0.003	4.014
*Cd200*	OX-2 membrane glycoprotein	0.365	8.688	0.002	3.968
*Gbp4*	Guanylate-binding protein 4	19.279	74.645	0.001	3.872
*Gbp1*	Guanylate-binding protein 1	18.157	68.355	0.000	3.765
*Bst2*	Bone marrow stromal antigen 2	5.948	26.738	0.031	3.746
*Ifit1*	Interferon-induced protein with tetratricopeptide repeats 1	18.342	68.266	0.001	3.722
*Isg15*	Ubiquitin-like protein ISG15	112.471	411.178	0.039	3.656
*Phf11*	PHD finger protein 11	1.918	13.647	0.030	3.557
*Gbp5*	Guanylate-binding protein 5	9.073	47.393	0.014	3.482
*Ifit2*	Interferon-induced protein with tetratricopeptide repeats 2	25.488	83.357	0.007	3.270
*Ifit3*	Interferon-induced protein with tetratricopeptide repeats 3	22.823	88.089	0.004	3.216
*Ptgs2*	Prostaglandin G/H synthase 2	1.107	20.992	0.038	3.161
*Iigp1*	Interferon-inducible GTPase 1	8.602	31.608	0.002	3.062
*Cd274*	Programmed cell death 1 ligand 1	3.463	19.962	0.024	2.883
*Gbp2*	Guanylate-binding protein 2	11.134	36.508	0.007	2.733
*H2-T23*	H-2 class I histocompatibility antigen, D-37 alpha chain	7.209	29.348	0.006	2.714
*Nt5c3a*	Cytosolic 5’-nucleotidase 3A	9.823	26.521	0.002	2.700
*Slc7a2*	Cationic amino acid transporter 2	0.832	12.178	0.004	2.439
*Sp110*	Sp110 nuclear body protein	11.318	27.038	0.005	2.389
*Tapbp*	Tapasin	165.935	380.833	0.006	2.295
*H2-Q8*	H-2 class I histocompatibility antigen, Q8 alpha chain	0.888	12.071	0.002	2.265
*Slc15a3*	Solute carrier family 15 member 3	4.673	20.979	0.014	2.245
*Icam1*	Intercellular adhesion molecule 1	31.871	70.840	0.007	2.223
*Isg20*	Interferon-stimulated gene 20 kDa protein	1.486	18.469	0.029	2.071
*Trex1*	Three-prime repair exonuclease 1	22.766	45.811	0.038	2.012
*Daxx*	Death domain-associated protein 6	3.396	19.414	0.020	1.906
*Rnaset2a; Rnaset2b*	Ribonuclease T2-A	2.402	9.037	0.038	1.881
*Sdc1*	Syndecan-1	2.376	12.898	0.025	1.810
*H2-L*	H-2 class I histocompatibility antigen, L-D alpha chain	217.562	379.962	0.002	1.746
*Tap1*	Antigen peptide transporter 1	20.946	36.059	0.013	1.721
*Serpina3k*	Serine protease inhibitor A3K	0.414	4.245	0.010	1.708
*Clic4*	Chloride intracellular channel protein 4	59.976	101.831	0.006	1.698
*Clec4e*	C-type lectin domain family 4 member E	0.873	8.762	0.007	1.672
*Il1rn*	Interleukin-1 receptor antagonist protein	22.857	37.083	0.018	1.622
*Tap2*	Antigen peptide transporter 2	37.013	55.981	0.026	1.513
*Nfkb2*	Nuclear factor NF-kappa-B p100 subunit	26.719	39.959	0.039	1.496
*Chmp4b*	Charged multivesicular body protein 4b	88.550	131.179	0.046	1.481
*Sema4d*	Semaphorin-4D	0.549	4.822	0.019	1.463
*Plxna1*	Plexin-A1	23.996	34.045	0.026	1.419
*Rnf114*	E3 ubiquitin-protein ligase RNF114	13.082	18.431	0.031	1.409
*Irgm1*	Immunity-related GTPase family M protein 1	62.808	87.582	0.034	1.394
*Mthfd2*	Bifunctional methylenetetrahydrofolate dehydrogenase/cyclohydrolase, mitochondrial	1.540	11.974	0.027	1.296
*Hspa5*	Endoplasmic reticulum chaperone BiP	402.346	512.956	0.030	1.275
*Src*	Proto-oncogene tyrosine-protein kinase Src	35.885	45.608	0.039	1.271
*Aldh1b1*	Aldehyde dehydrogenase X, mitochondrial	20.697	26.002	0.022	1.256
*Gyg1*	Glycogenin-1	43.732	54.653	0.013	1.250
*Oat*	Ornithine aminotransferase, mitochondrial	45.390	56.139	0.039	1.237
*Tlr2*	Toll-like receptor 2	20.473	25.201	0.040	1.231
*Nampt*	Nicotinamide phosphoribosyltransferase	102.911	125.617	0.027	1.221
*Tgm2*	Protein-glutamine gamma-glutamyltransferase 2	59.923	71.772	0.020	1.198
*Pdia3*	Protein disulfide-isomerase A3	427.181	508.180	0.013	1.190
*Dlat*	Dihydrolipoyllysine-residue acetyltransferase component of pyruvate dehydrogenase complex, mitochondrial	82.697	98.320	0.038	1.189
*Hnrnpm*	Heterogeneous nuclear ribonucleoprotein M	150.866	174.278	0.033	1.155
*Sec11a*	Signal peptidase complex catalytic subunit SEC11A	23.882	27.462	0.038	1.150
*Hsp90ab1*	Heat shock protein HSP 90-beta	811.190	928.346	0.024	1.144
*Hsp90b1*	Endoplasmin	904.794	1006.715	0.024	1.113
*Capn2*	Calpain-2 catalytic subunit	107.346	90.642	0.010	0.844
*Gpaa1*	Glycosylphosphatidylinositol anchor attachment 1 protein	22.174	17.066	0.042	0.770
*Cpt1a*	Carnitine O-palmitoyltransferase 1, liver isoform	37.425	28.763	0.003	0.769
*Lrp1*	Prolow-density lipoprotein receptor-related protein 1	102.596	77.790	0.003	0.758
*Aqr*	RNA helicase aquarius	16.361	12.218	0.048	0.747
*Ddb1*	DNA damage-binding protein 1	30.754	22.739	0.038	0.739
*Ubxn6*	UBX domain-containing protein 6	14.477	10.133	0.038	0.700
*Rpl22l1*	60S ribosomal protein L22-like 1	27.692	19.173	0.040	0.692
*Glg1*	Golgi apparatus protein 1	34.516	22.497	0.009	0.652
*Apoe*	Apolipoprotein E	192.407	96.859	0.024	0.503
*Cd300ld3*	CMRF35-like molecule 3	17.868	8.905	0.006	0.498
*Lpl*	Lipoprotein lipase	35.613	13.226	0.027	0.446
*Csf1r*	Macrophage colony-stimulating factor 1 receptor	43.767	15.612	0.027	0.428
*Colec12*	Collectin-12	22.477	8.953	0.002	0.398

* The statistical significance of these proteins was determined using Welch’s *t*-test with a significance threshold of *q* < 0.05, corrected for *p*-values by Welch’s *t*-test.

## Data Availability

The data presented in this study are openly available in ProteomeXchange at https://www.proteomexchange.org/, reference number PXD046763 for the proteomic data of NS and HS and reference number PXD046766 for the proteomic data of Ssa1-treated and untreated BMMs. The raw data supporting the conclusions of this article will be made available by the authors on request.

## Supplementary Materials

The following supporting information can be downloaded at: https://www.mdpi.com/article/10.3390/cells13020127/s1, Figure S1. Difference in protein pattern between NS and HS. Figure S2. Recombinant *C. albicans* Ssa1, and its effect on cytokine production and viability of RAW 264.7 cells. Figure S3. Construction of *C. albicans* Ssa1^−^ mutant by CRISPR/Cas9 method. Figure S4. Figure 2B Zoom-in. Figure S5. Figure 2C Zoom-in. Table S1. Primers for gene expression quantified by RT-qPCR. Table S2. Oligomers and primers for Ssa1^−^ construction. Table S3. Protein identified in NS and HS of *C. albicans.* Table S4. List of proteins that were significantly less abundant in hypoxic secretome (*p* < 0.05). Table S5. Significant differential proteins between Ssa1-treated and untreated BMMs. Table S6. GO enrichment of the significantly up-regulated and down-regulated proteins in Ssa1-treated mouse BMMs.
