# Supplementary material for: Heat Shock Protein SSA1 Enriched in Hypoxic Secretome of Candida albicans Exerts an Immunomodulatory Effect via Regulating Macrophage Function
Source: Cells. 2024 Jan 10;13(2):127. doi: 10.3390/cells13020127 (PMC10814802; doi:10.3390/cells13020127)
Supplement: Supplementary file 1 [file cells-13-00127-s001.zip › Figure S1.pdf]

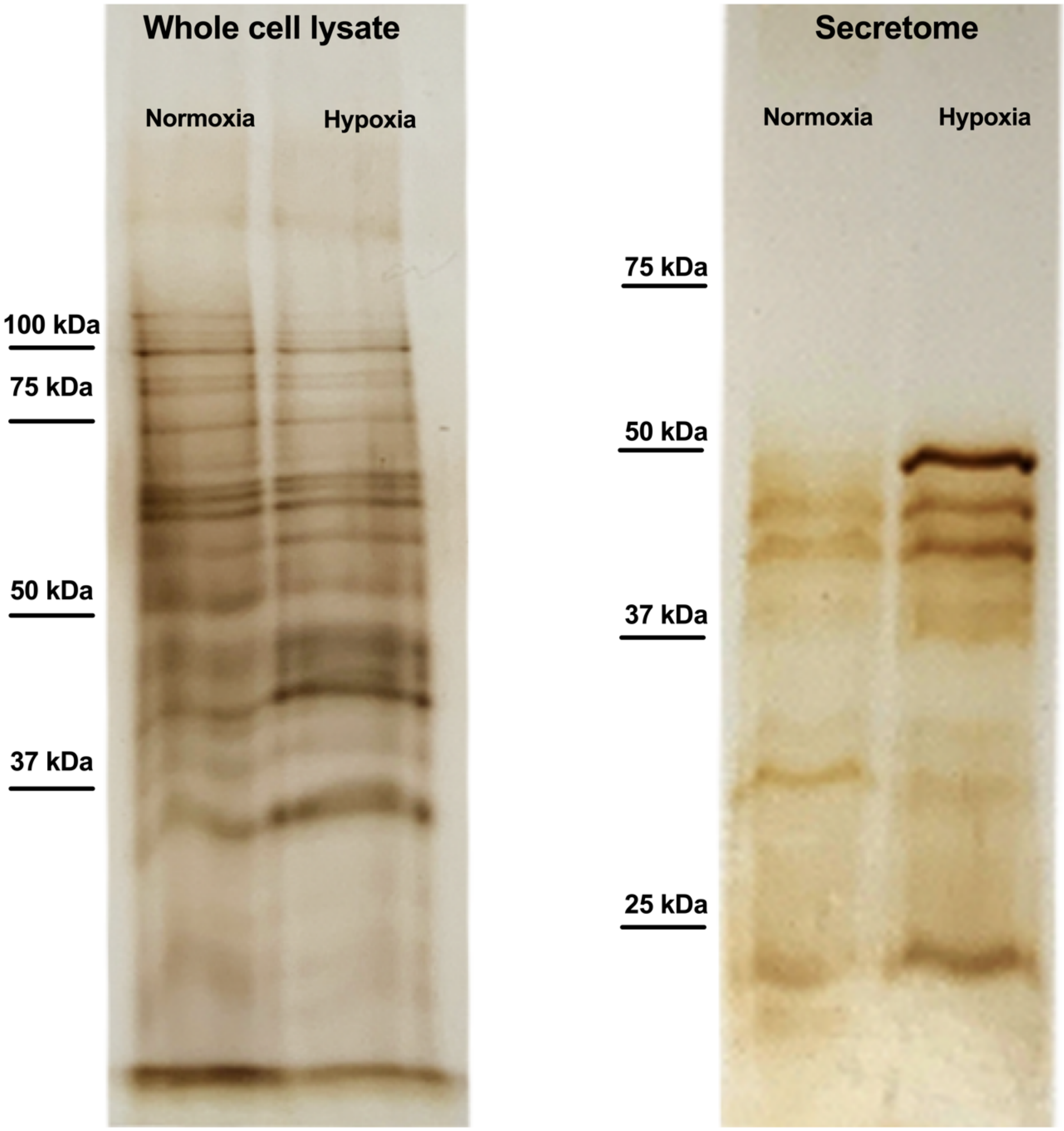

Figure S1: Difference in protein pattern between NS and HS. Five μg protein of NS and HS was applied to 10% SDS-polyacrylamide gel. Yeast cell lysates from normoxic and hypoxic culture condition were also applied to 10% gel. After electrophoresis, the gels were stained using Silver Stain II Kit (Wako) according to the manufacturer's protocol.
