# Supplementary material for: Heat Shock Protein SSA1 Enriched in Hypoxic Secretome of Candida albicans Exerts an Immunomodulatory Effect via Regulating Macrophage Function
Source: Cells. 2024 Jan 10;13(2):127. doi: 10.3390/cells13020127 (PMC10814802; doi:10.3390/cells13020127)
Supplement: Supplementary file 1 [file cells-13-00127-s001.zip › Figure S2.pdf]

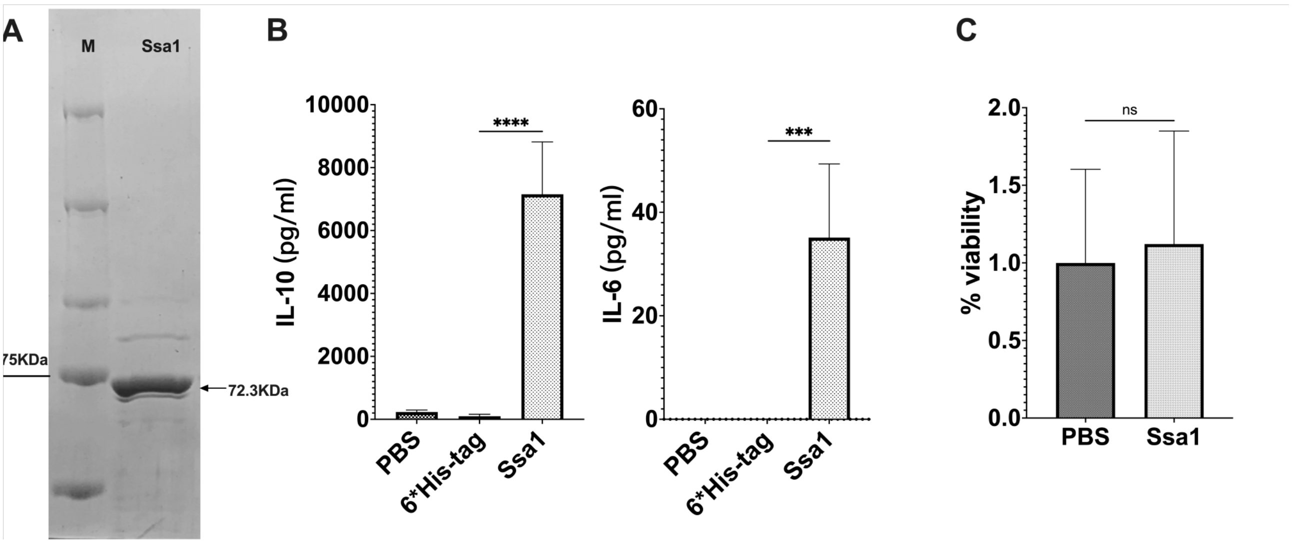

Figure S2. Recombinant *C. albicans* Ssa1 (A), and its effect on cytokine production (B) and viability (C) of RAW 264.7 cells. (A) Purified recombinant Ssa1 on 8% SDS-polyacrylamide gel with CBB-staining. (His)6-tagged Ssa1 from *E. coli* lysate was purified, and lipopolysaccharide was removed as described in the Materials and Methods. From 1.2 L of *E. coli* culture, 7.36 mg of recombinant Ssa1 protein was obtained. The estimated sized of (His)6-tagged Ssa1 was 72.3 kDa. **(B)** Cytokine production of RAW 264.7 cells after stimulated by recombinant Ssa1. RAW 264.7 cells (1×10^6 cells) were seeded in 24-well plates and incubated with or without 5 μg recombinant Ssa1 for 48 h, and the production IL-10 and IL-6 was determined by ELISA. To examine neither 6 histamine residues nor residual lipopolysaccharide affect this cytokine response, a 2.3 kDa (His)6-containing protein from the pET-15b plasmid was prepared by the same procedure and 0.5 μg of this protein used as a control. As the molecular weight of the (His)6-protein control (2.3 kDa) is 31-fold smaller than that of (His)6-tagged Ssa1 (72.3 kDa), 0.5 μg (His)6-protein control (217.39 pmol) was three times higher in molar mass than 5 μg (His)6-tagged Ssa1 (69.16 pmol). There was a significant difference in the production of IL-10 and IL-6 between (His)6-protein control and (His)6-tagged Ssa1 (****: $P < 0.0001$, ***: $P < 0.0002$, statistical analysis by unpaired Student's *t*-test, n = 6). Moreover, no significant differences in cytokine production between PBS group and (His)6-protein control group were found, indicating that the cytokine response was due to Ssa1, but not 6 histidine residues or lipopolysaccharide. **(C)** Recombinant Ssa1 does not affect cell viability of mouse macrophages. RAW 264.7 cells (1×10^4 cells/well) were seeded in a 96-well plate with or without 0.5 μg Ssa1. After 24 h of incubation, the culture supernatant in each well was replaced with 100 μL of fresh medium containing 10 μL of cell proliferation reagent (WST1; Roche). After color development, the absorbance at 420 nm was measured using the absorbance at 600 nm as a reference wavelength. Cell-free medium was used as blank, and the absorbance of RAW 264.7 cells without Ssa1 was calculated as 100% viability (ns: not significant, statistical analysis by unpaired Students' *t*-test, n = 9 from 3 independent experiments).
