# Supplementary material for: Heat Shock Protein SSA1 Enriched in Hypoxic Secretome of Candida albicans Exerts an Immunomodulatory Effect via Regulating Macrophage Function
Source: Cells. 2024 Jan 10;13(2):127. doi: 10.3390/cells13020127 (PMC10814802; doi:10.3390/cells13020127)
Supplement: Supplementary file 1 [file cells-13-00127-s001.zip › Figure S3.pdf]

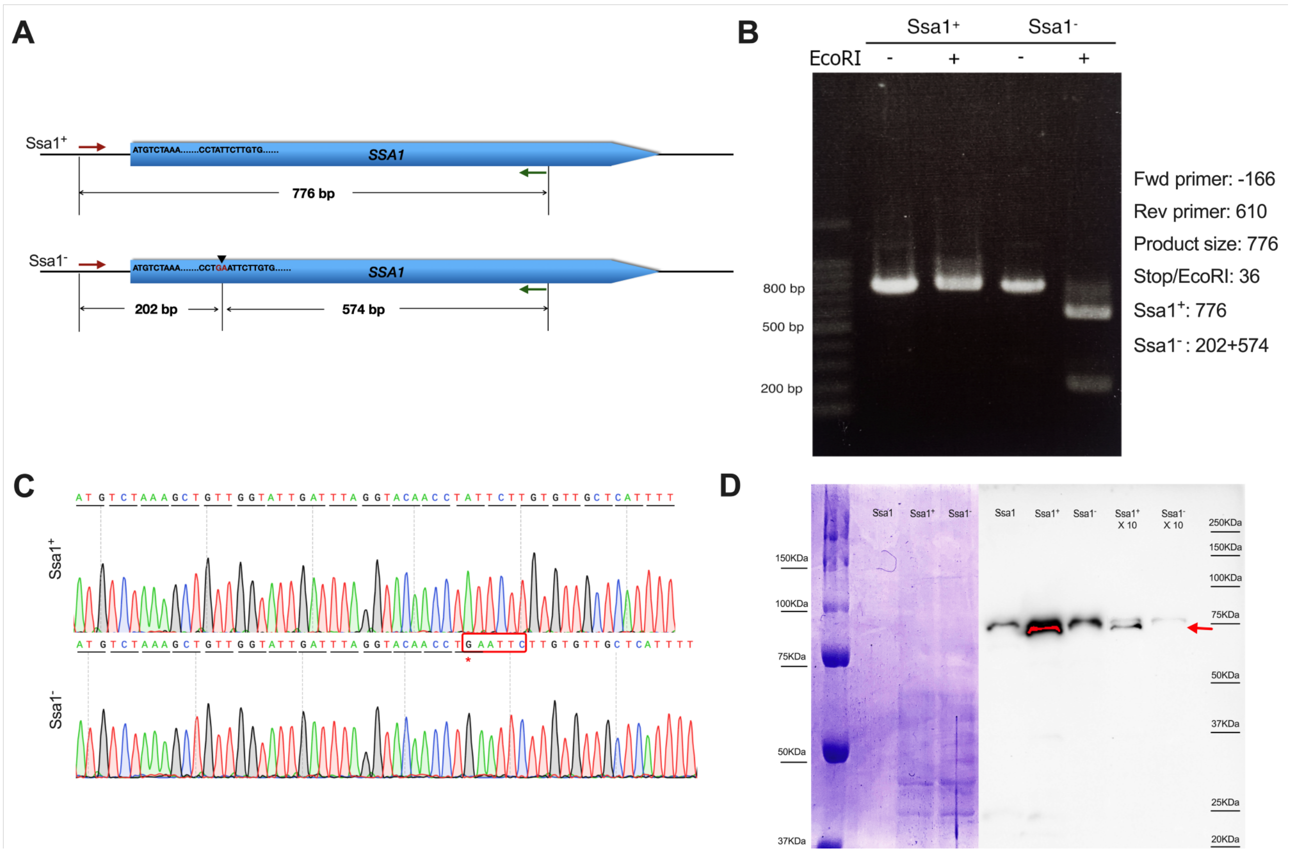

Figure S3: Construction of *C. albicans* Ssa1^{-} mutant by CRISPR/Cas9 method. (A) Scheme of *SSA1* gene in *C. albicans* NBRC 1385 wild type (Ssa^{+}) and Ssa1^{-} mutant. Based on the repair template sequence (Supplementary Table 2), nucleotides "G" and "A" were inserted into *SSA1* gene to create a stop codon and EcoRI recognition site. To screen Ssa1^{-} mutant, a 776-bp PCR product was amplified using checking primers (Supplementary Table 2), and the PCR product from the Ssa1^{-} mutant was digested with EcoRI to generate 202 bp and 574 bp DNA fragments. **(B)** Presence of EcoRI recognition site in the *SSA1* gene of *C. albicans* Ssa1^{-} was demonstrated by electrophoresis on a 1% agarose gel. **(C)** The presence of stop codon and EcoRI recognition site in the *SSA1* gene of *C. albicans* Ssa1^{-} was confirmed by sequencing. Insertion of nucleotides "G" and "A" (orange square) into the *SSA1* gene of *C. albicans* Ssa1^{-} indicates that a stop codon was generated, and only N-terminal 12 residues from the total 656 amino acids of the Ssa1 protein were translated. **(D)** Confirmation of *C. albicans* Ssa1^{-} mutant by Western blot analysis. The Ssa1 positive band from the recombinant Ssa1 and *C. albicans* Ssa1^{+} lysate (red arrow) but not *C. albicans* Ssa1^{-} lysate was detected using anti-Ssa1 polyclonal antibodies in mouse serum (prepared in our laboratory) and HRP-conjugated anti-mouse IgG (Abcam, Tokyo, Japan). To prepare anti-Ssa1 polyclonal antibodies, BALB/c mice (with 7 weeks of age) were subcutaneously immunized with 10 µg recombinant Ssa1 mixed with Imject® Alum (Thermo Scientific) on Day 0 and 14. On Day 28, mice were anesthetized, and whole blood was taken. Whole blood was incubated at 37°C for 30 min and then centrifuged at 800×*g* for 15 min to obtained serum. (Left) Equal volume of whole cell lysate from *C. albicans* wild type (Ssa^{+}) and Ssa1^{-} mutant were loaded on 8% SDS-polyacrylamide gel with CBB-staining.
