# Supplementary material for: Heat Shock Protein SSA1 Enriched in Hypoxic Secretome of Candida albicans Exerts an Immunomodulatory Effect via Regulating Macrophage Function
Source: Cells. 2024 Jan 10;13(2):127. doi: 10.3390/cells13020127 (PMC10814802; doi:10.3390/cells13020127)
Supplement: Supplementary file 1 [file cells-13-00127-s001.zip › Table S1.pdf]

Table S1 Primers for gene expression quantified by RT-qPCR.

Gene	Primer	Sequence
CD86	Forward	5’-ACGATGGACCCCAGATGCACCA-3’
	Reverse	5’-GCGTCTCCACGGAAACAGCA-3’
CD11c	Forward	5’-TGGGGTTTGTTTCTTGTCTTG-3’
	Reverse	5’-GCCTGTGTGATAGCCACATTT-3’
Arg1	Forward	5’-GGTCCAGAAGAATGGAAGAGTCAG-3’
	Reverse	5’-TGGCAGATATGCAGGGAGTCACC-3’
CD206	Forward	5’-GGTTCGGGATTGTGGAGCAGAT-3’
	Reverse	5’-CACAGACGGCTCCATTTGCATT-3’
GAPDH	Forward	5’-TGAAGGTCGGTGTGAACGGATTTGG-3’
	Reverse	5’-ACGACATACTCAGCACCGGCCTCAC-3’
CCL1	Forward	5’-GCTTACGGTCTCCAATAGCTGC-3’
	Reverse	5’-GCTTTCTCTACCTTTGTTCAGCC-3’
LIGHT (TNFSF14)	Forward	5’-GGCTGGTTTCTCCTGAGACTGC-3’
	Reverse	5’-CCCCATCATGATACGTCAAGCC-3’
