# Supplementary material for: Heat Shock Protein SSA1 Enriched in Hypoxic Secretome of Candida albicans Exerts an Immunomodulatory Effect via Regulating Macrophage Function
Source: Cells. 2024 Jan 10;13(2):127. doi: 10.3390/cells13020127 (PMC10814802; doi:10.3390/cells13020127)
Supplement: Supplementary file 1 [file cells-13-00127-s001.zip › Table S2.pdf]

Table S2 Oligomers and primers for Ssa1^{-} construction

Oligomer/primer	Sequence
guide-RNA oligos F	5'-ATTTGAAAATGAGCAACACAAGAATG-3'
guide-RNA oligos R	5'-AAAACATTCTTGTGTTGCTCATTTTC-3'
Repair template primer upstream F	5'-CTCTTTCTAGTTTATTCCAATG-3'
Repair template primer upstream R	5'-AATGAGCAACACAAGAAT**TCA**GGTTGT-3'
Repair template primer downstream F	5'-ATTGATTTAGGTACAACC**TGA**ATTCTTG-3'
Repair template primer downstream R	5'-AAATGGGAAATGTTTAGCATCA-3'
Checking primer F	5'-GTACCACCACCCAAATCG-3'
Checking primer R	5'-CCTTAAATCACCTTTTCAACAATTTTCC-3'
Sequencing F	5'-TATTCTATTCTATTTCTTCTTTAC-3'
Sequencing R	5'-GGATCATCAAATTTCTCCCAA-3'

Underlines: EcoRI recognition site in repair template
Bold font: Stop codon
