# Supplementary material for: Heat Shock Protein SSA1 Enriched in Hypoxic Secretome of Candida albicans Exerts an Immunomodulatory Effect via Regulating Macrophage Function
Source: Cells. 2024 Jan 10;13(2):127. doi: 10.3390/cells13020127 (PMC10814802; doi:10.3390/cells13020127)
Supplement: Supplementary file 1 [file cells-13-00127-s001.zip › Table S4.pdf]

Table S4. List of proteins that were significantly less abundant in hypoxic secretome ($P < 0.05$)

Gene ID	Protein Name	*P*-value	Fold Change (Hypoxic vs Normoxic)
HHF1	Histone H4	0.01428	0
CAT1	Peroxisomal catalase	0.04663	0.0102965
PET9	ADP/ATP translocase	0.04014	0.019962
RPS19A	Ribosomal 40S subunit protein S19A	0.00192	0.0235732
RPL38	Ribosomal 60S subunit protein L38	0.00341	0.0256623
TMA19	Translationally-controlled tumor protein homolog	0.00691	0.0271787
RPS20	Ribosomal 40S subunit protein S20	0.00002	0.0454775
RAD23	UV excision repair protein RAD23	0.01059	0.0473624
CAALFM_C502400WA	Acyl-protein thioesterase 1	0.02943	0.0488435
COX5	Cytochrome c oxidase subunit Va	0.01808	0.0524154
DUT1	Deoxyuridine 5'-triphosphate nucleotidohydrolase	0.00324	0.0536025
MDH1-1	Malate dehydrogenase	0.00004	0.0549651
COX2	Cytochrome c oxidase subunit 2	0.01501	0.0555613
DPH5	Diphthine methyl ester synthase 1	0.01436	0.0682456
CAALFM_CR03020CA	5'-Deoxynucleotidase	0.01800	0.0921407
FUM12	Fumarate hydratase	0.00219	0.0961438
orf19.5783	Protein-arginine N-methyltransferase	0.00406	0.0976128
SSC1	Heat shock protein SSC1, mitochondrial	0.00813	0.0995035
THS1	Threonyl-tRNA synthetase	0.00353	0.1027321
POR1	Mitochondrial outer membrane protein	0.00181	0.1062212
LOS1	Exportin-T	0.03128	0.1085245
SSA2	Heat shock protein SSA2	0.00014	0.1131992
ATP2	ATP synthase subunit beta	0.00018	0.1218883
RPS12	40S ribosomal protein S12	0.04509	0.1309219
NPT1	Nicotinate phosphoribosyltransferase	0.04194	0.1332343
orf19.2489	Uncharacterized protein	0.01524	0.1341046
ALD5	Aldehyde dehydrogenase (NAD(P)(+))	0.04257	0.138377
THR1	Homoserine kinase	0.01069	0.1418983
LAT1	Acetyltransferase component of pyruvate dehydrogenase complex	0.03934	0.145565
MIC60	MICOS complex subunit MIC60	0.00737	0.148982
orf19.6717	Putative serine hydrolase	0.00227	0.1496993
PDB1	Pyruvate dehydrogenase E1 component subunit beta	0.00650	0.1541602
RNA1	GTPase-activating protein	0.00387	0.1609203
HGT6	Hexose transporter	0.00918	0.1631077
ARD	Ardp	0.00699	0.1679522
CAALFM_CR10140WA	Uncharacterized protein	0.00238	0.1851241
orf19.5627	Uncharacterized protein	0.03996	0.1932374

Table S4. List of proteins that were significantly less abundant in hypoxic secretome ($P < 0.05$)

CHS3	Chitin synthase	0.00467	0.1976797
ERG10	Acetyl-CoA C-acetyltransferase	0.03883	0.1977768
PNP1	Purine nucleoside phosphorylase	0.01285	0.2027644
ERO1	ER oxidoreductin	0.01050	0.2045617
CAALFM_CR03520CA	Uncharacterized protein	0.04243	0.2060871
orf19.4149.1	Ribosomal 40S subunit protein S11A	0.00556	0.2067683
AIP2	D-lactate dehydrogenase	0.00707	0.2126529
GLK1	Phosphotransferase	0.01040	0.2144066
MRPL4	54S ribosomal protein L4, mitochondrial	0.00126	0.220519
TIF5	Translation initiation factor eIF5	0.01271	0.2218943
CIT1	Citrate synthase	0.01295	0.2321603
RPL27A	60S ribosomal protein L27	0.00221	0.2451604
TUP1	Transcriptional repressor TUP1	0.00382	0.2475561
PGM2	Phosphoglucomutase	0.02810	0.2612159
PHM7	Phm7p	0.00061	0.2625286
RPS6A	40S ribosomal protein S6	0.00666	0.2648646
orf19.6701	Prolyl-tRNA synthetase	0.03186	0.2673893
GLN4	Glutaminyl-tRNA synthetase	0.01776	0.2852747
RPL32	Ribosomal 60S subunit protein L32	0.01907	0.2936814
GUA1	GMP synthase [glutamine-hydrolyzing]	0.00866	0.3205714
orf19.6559	Transcription factor TFIIIC subunit	0.03934	0.3323373
CYS3	Cystathionine gamma-lyase	0.02218	0.3369011
CAALFM_CR03740CA	Flavin mononucleotide kinase 1	0.00929	0.3388528
URA2	Aspartate carbamoyltransferase	0.00022	0.3638344
PIL1	Lipid-binding protein	0.02135	0.3686599
PDI1	Protein disulfide-isomerase	0.01568	0.374841
BNA5	Kynureninase	0.03719	0.383384
ARC1	Arc1p	0.03805	0.3857364
RIB3	3,4-Dihydroxy-2-butanone 4-phosphate synthase	0.04770	0.390528
orf19.3053	Uncharacterized protein	0.01348	0.4065866
ATP4	ATP synthase subunit 4	0.00952	0.4099336
LKH1	Leucine aminopeptidase 2	0.01740	0.4869792
RPS24	40S ribosomal protein S24	0.00904	0.501866
SAP2	Candidapepsin-2	0.04047	0.5108188
RPS22A	40S ribosomal protein S22-A	0.00245	0.5465655
ARF3	Arf3p	0.03362	0.5566354
KRE30	ATP-binding cassette family ATPase	0.02218	0.5724679
