# Supplementary material for: Heat Shock Protein SSA1 Enriched in Hypoxic Secretome of Candida albicans Exerts an Immunomodulatory Effect via Regulating Macrophage Function
Source: Cells. 2024 Jan 10;13(2):127. doi: 10.3390/cells13020127 (PMC10814802; doi:10.3390/cells13020127)
Supplement: Supplementary file 1 [file cells-13-00127-s001.zip › Table S6.pdf]

**Table S6. GO enrichment of the significantly up-regulated and down-regulated proteins in Ssa1-treated mouse BMMs**

| Function | Group Genes |
|---|---|
| signaling receptor binding | *Apoe, Cd86, H2-Q8, H2-T23, Hsp90ab1, Hsp90b1, Icam1, Il1rn, Isg15, Lpl, Lrp1, Nampt, Pdia3, Sema4d, Src, Tap1, Tap2, Tlr2* |
| protein-containing complex binding | *Apoe, Capn2, Clic4, Colec12, Ddb1, Gbp2, H2-Q8, H2-T23, Hspa5, Icam1, Isg15, Lpl, Lrp1, Src, Tap1, Tap2, Tapbp, Tlr2, Trex1* |
| establishment of protein localization | *Apoe, Cd200, Chmp4b, Cpt1a, Gbp4, Hnrnpm, Hsp90ab1, Hsp90b1, Hspa5, Il1rn, Lrp1, Ptgs2, Rsad2, Slc15a3, Src, Tap1, Tap2, Tlr2, Trex1* |
| binding | *Apoe, Aqr, Bst2, Capn2, Cd200, Cd274, Cd300ld3, Cd40, Cd86, Chmp4b, Clec4e, Clic4, Cmpk2, Colec12, Cpt1a, Csf1r, Daxx, Ddb1, Dlat, Gbp2, Gbp2b, Gbp4, Gbp5, Glg1, Gpaa1, Gyg, H2-Q8, H2-T23, Herc6, Hnrnpm, Hsp90ab1, Hsp90b1, Hspa5, Icam1, Ifi205, Ifit1, Ifit2, Ifit3, Iigp1, Il18bp, Il1rn, Irgm1, Isg15, Isg20, Lpl, Lrp1, Mov10, Mthfd2, Nampt, Nfkb2, Nt5c3, Oasl1, Oat, Pdia3, Phf11, Plxna1, Ptgs2, Rnaset2a, Rnaset2b, Rnf114, Rpl22l1, Rsad2, Sdc1, Sema4d, Serpina3k, Sp110, Src, Tap1, Tap2, Tapbp, Tgm2, Tlr2, Trex1* |
| protein binding | *Apoe, Bst2, Capn2, Cd200, Cd274, Cd300ld3, Cd40, Cd86, Chmp4b, Clec4e, Clic4, Cpt1a, Csf1r, Daxx, Ddb1, Dlat, Gbp2, Gbp2b, Gbp4, Gbp5, Glg1, Gyg, H2-Q8, H2-T23, Herc6, Hnrnpm, Hsp90ab1, Hsp90b1, Hspa5, Icam1, Ifi205, Ifit1, Ifit2, Ifit3, Iigp1, Il18bp, Il1rn, Irgm1, Isg15, Lpl, Lrp1, Mov10, Nampt, Nfkb2, Oat, Pdia3, Plxna1, Ptgs2, Rsad2, Sdc1, Sema4d, Serpina3k, Src, Tap1, Tap2, Tapbp, Tlr2, Trex1* |
| viral process | *Apoe, Bst2, Chmp4b, Csf1r, Ddb1, Hsp90ab1, Icam1, Isg15, Isg20, Oasl1, Rsad2* |
| hydrolase activity, acting on ester bonds | *Cd86, Hsp90ab1, Hsp90b1, Isg20, Lpl, Lrp1, Nt5c3, Oasl1, Rnaset2a, Rnaset2b, Sema4d, Src, Trex1* |
| identical protein binding | *Apoe, Bst2, Chmp4b, Cpt1a, Csf1r, Daxx, Dlat, Gbp2, Gbp2b, Gbp4, Gbp5, Gyg, H2-Q8, H2-T23, Hsp90ab1, Ifit3, Iigp1, Lpl, Nampt, Oat, Pdia3, Ptgs2, Sdc1, Sema4d, Tap1, Tap2, Tlr2, Trex1* |
| hydrolase activity, acting on acid anhydrides | *Apoe, Aqr, Cd40, Clec4e, Cmpk2, Csf1r, Gbp2, Gbp2b, Gbp4, Gbp5, Gpaa1, Hsp90ab1, Hsp90b1, Hspa5, Icam1, Iigp1, Irgm1, Lpl, Mov10, Plxna1, Sema4d, Src, Tap1, Tap2, Tgm2, Tlr2, Trex1* |
| hydrolase activity | *Acod1, Aldh1b1, Apoe, Aqr, Bst2, Capn2, Cd200, Cd40, Cd86, Cmpk2, Cpt1a, Csf1r, Daxx, Dlat, Gbp2, Gbp2b, Gbp4, Gbp5, Gpaa1, Gyg, Herc6, Hsp90ab1, Hsp90b1, Hspa5, Icam1, Iigp1, Il1rn, Irgm1, Isg20, Lpl, Lrp1, Mov10, Mthfd2, Nampt, Nt5c3, Oasl1, Oat, Pdia3, Plxna1, Ptgs2, Rnaset2a, Rnaset2b, Rnf114, Rsad2, Sec11a, Sema4d, Serpina3k, Src, Tap1, Tgm2, Tlr2, Trex1* |
| regulation of response to external stimulus | *Acod1, Apoe, Cd200, Cd274, Cd40, Clec4e, Csf1r, Gbp4, Gbp5, Hspa5, Ifi205, Irgm1, Isg15, Lpl, Lrp1, Oasl1, Ptgs2, Rsad2, Sema4d, Slc7a2, Src, Tap1, Tap2, Tlr2, Trex1* |
| cellular catabolic process | *Aldh1b1, Apoe, Capn2, Chmp4b, Cpt1a, Ddb1, Herc6, Hnrnpm, Hsp90ab1, Hsp90b1, Hspa5, Iigp1, Irgm1, Isg15, Isg20, Lpl, Lrp1, Mov10, Nampt, Nt5c3, Oat, Rnaset2a, Rnaset2b, Rnf114, Tlr2, Trex1, Ubxn6* |
| adaptive immune response | *Acod1, Apoe, Bst2, Cd200, Cd274, Cd40, Cd86, Clec4e, Csf1r, Gbp5, H2-T23, Icam1, Ifi205, Il18bp, Irgm1, Lrp1, Nampt, Nfkb2, Rsad2, Slc7a2, Tap1, Tap2, Tlr2, Trex1* |
| regulation of cytokine production | *Acod1, Apoe, Bst2, Capn2, Cd200, Cd274, Cd300ld3, Cd40, Cd86, Clec4e, Colec12, Csf1r, Daxx, Gbp4, Gbp5, Glg1, H2-T23, Hsp90ab1, Hspa5, Icam1, Ifi205, Il1rn, Irgm1, Isg15, Lpl, Lrp1, Oasl1, Pdia3, Ptgs2, Rsad2, Sema4d, Slc15a3, Slc7a2, Src, Tap1, Tap2, Tapbp, Tgm2, Tlr2, Trex1* |
| immune response | *Acod1, Apoe, Bst2, Cd200, Cd274, Cd300ld3, Cd40, Cd86, Clec4e, Colec12, Csf1r, Daxx, Gbp2, Gbp2b, Gbp4, Gbp5, Glg1, H2-L, H2-Q8, H2-T23, Herc6, Hsp90ab1, Hspa5, Icam1, Ifi205, Ifit1, Ifit2, Ifit3, Iigp1, Il18bp, Il1rn, Irgm1, Isg15, Isg20, Lpl, Lrp1, Nampt, Nfkb2, Oasl1, Pdia3, Ptgs2, Rnaset2a, Rnaset2b, Rsad2, Sema4d, Slc15a3, Slc7a2, Sp110, Src, Tap1, Tap2, Tapbp, Tgm2, Tlr2, Trex1* |
| defense response | *AA467197, Acod1, Apoe, Bst2, Capn2, Cd200, Cd274, Cd300ld3, Cd40, Cd86, Chmp4b, Clec4e, Clic4, Cmpk2, Colec12, Cpt1a, Csf1r, Daxx, Ddb1, Gbp2, Gbp2b, Gbp4, Gbp5, Glg1, H2-L, H2-Q8, H2-T23, Herc6, Hnrnpm, Hsp90ab1, Hsp90b1, Hspa5, Icam1, Ifi205, Ifit1, Ifit2, Ifit3, Iigp1, Il18bp, Il1rn, Irgm1, Isg15, Isg20, Lpl, Lrp1, Mov10, Nampt, Nfkb2, Nt5c3, Oasl1, Pdia3, Plxna1, Ptgs2, Rnaset2a, Rnaset2b, Rsad2, Sdc1, Sema4d, Serpina3k, Slc15a3, Slc7a2, Sp110, Src, Tap1, Tap2, Tapbp, Tgm2, Tlr2, Trex1, Ubxn6* |
| response to organic substance | *Acod1, Apoe, Bst2, Capn2, Cd200, Cd274, Cd300ld3, Cd40, Cd86, Chmp4b, Clec4e, Clic4, Cmpk2, Colec12, Cpt1a, Csf1r, Daxx, Ddb1, Gbp2, Gbp2b, Gbp4, Gbp5, Glg1, H2-T23, Hnrnpm, Hsp90ab1, Hsp90b1, Hspa5, Icam1, Ifi205, Ifit1, Ifit2, Ifit3, Iigp1, Il1rn, Irgm1, Isg15, Lpl, Lrp1, Mov10, Nampt, Nfkb2, Oasl1, Pdia3, Plxna1, Ptgs2, Rsad2, Sdc1, Sema4d, Serpina3k, Slc15a3, Sp110, Src, Tap2, Tgm2, Tlr2, Trex1, Ubxn6* |
| regulation of immune response | *Acod1, Aldh1b1, Apoe, Aqr, Bst2, Capn2, Cd200, Cd274, Cd300ld3, Cd40, Cd86, Chmp4b, Clec4e, Clic4, Cmpk2, Colec12, Cpt1a, Csf1r, Daxx, Ddb1, Dlat, Gbp2, Gbp2b, Gbp4, Gbp5, Glg1, Gpaa1, Gyg, H2-T23, Herc6, Hnrnpm, Hsp90ab1, Hsp90b1, Hspa5, Icam1, Ifi205, Ifit1, Ifit2, Ifit3, Iigp1, Il1rn, Irgm1, Isg15, Isg20, Lpl, Lrp1, Mov10, Mthfd2, Nampt, Nfkb2, Nt5c3, Oasl1, Oat, Pdia3, Plxna1, Ptgs2, Rnaset2a, Rnaset2b, Rnf114, Rpl22l1, Rsad2, Sdc1, Sec11a, Sema4d, Serpina3k, Slc15a3, Slc7a2, Sp110, Src, Tap1, Tap2, Tapbp, Tgm2, Tlr2, Trex1, Ubxn6* |
