# Supplementary figures and images for: Heat Shock Protein SSA1 Enriched in Hypoxic Secretome of Candida albicans Exerts an Immunomodulatory Effect via Regulating Macrophage Function
Source: Cells. 2024 Jan 10;13(2):127. doi: 10.3390/cells13020127 (PMC10814802; doi:10.3390/cells13020127)

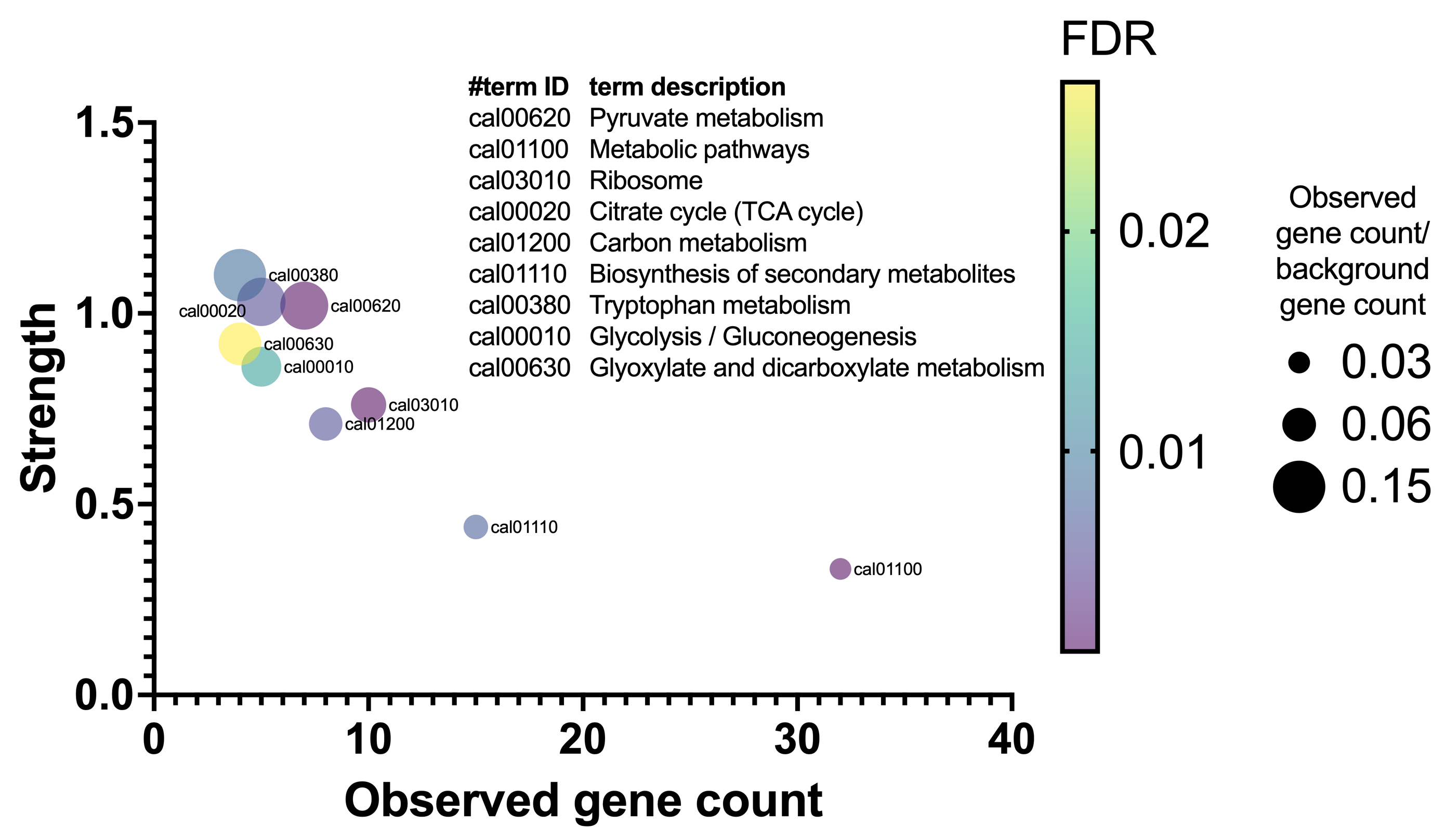

Supplement: Supplementary file 1 [file cells-13-00127-s001.zip › Figure S4. Fig2B Zoom-in.png]

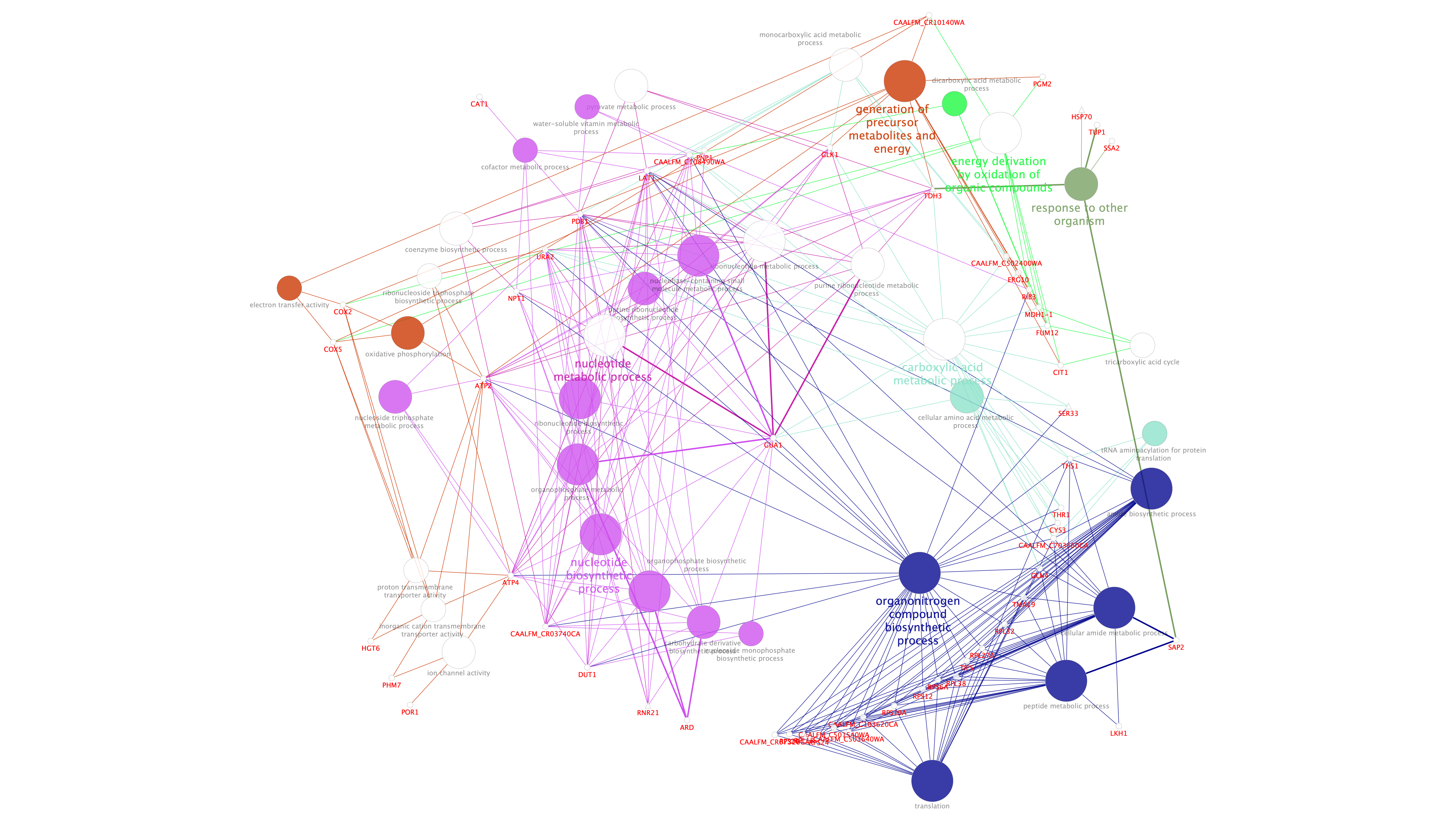

Supplement: Supplementary file 1 [file cells-13-00127-s001.zip › Figure S5. Fig2C Zoom-in.png]
